# Proteome‐Wide Target Identification Using Reactive Metallo‐Scaffolds (*r*‐mS): A Platform for Metallodrug Discovery

**DOI:** 10.1002/anie.8867549

**Published:** 2026-07-10

**Authors:** Jessica E. Waters, Harry Wilders, George S. Biggs, Mika Kintzel, Emma E. Cawood, Jonathan Bailey, Sarah Maslen, Yew Mun Yip, Mason Wakley, Ioannis G. Riziotis, Thomas W. Rees, Joanna Redmond, J. Mark Skehel, David House, Jacob Bush, Jeannine Hess

**Affiliations:** ^1^ The Biological Inorganic Chemistry Laboratory The Francis Crick Institute London UK; ^2^ Department of Chemistry King's College London London UK; ^3^ Crick‐GSK Biomedical Linklabs Stevenage Hertfordshire UK; ^4^ Proteomics Science Technology Platform The Francis Crick Institute London UK; ^5^ Chemical Biology Science Technology Platform The Francis Crick Institute London UK

**Keywords:** bioinorganic chemistry, cysteine targeting, PRMT1, proteomics, reactive metallo scaffolds

## Abstract

Metal complexes offer unique opportunities as scaffolds in chemical biology and drug discovery, with tunable geometries, modular coordination environments, and structural features not readily accessible with organic molecules. Here, we introduce reactive metallo‐scaffolds (***r*‐mS**) as a class of metal complexes designed to map ligandable cysteines across the mammalian proteome. These covalent warhead‐bearing metal complexes use the metal centre and ligand architecture to modulate cysteine engagement, with cysteine labelling occurring through the electrophilic chloroacetamide warhead. Using chemoproteomics, we profiled a focused *r*‐mS series in HEK293T lysate, identifying novel cysteine ligandability and demonstrating how metal identity, arene substituents and overall molecular topography govern cysteine reactivity and proteome‐wide targeting. Among the series screened, ***r*‐mS‐2** emerged as the most productive scaffold, which engaged cysteine 119 within the functionally relevant SAM‐binding domain of PRMT1. This interaction was validated by intact protein LC‐MS and was determined to functionally inhibit the activity of PRMT1. Structural modelling and docking provided insights into the molecular basis of binding, which implied π‐stacking and electrostatic complementarity in driving covalent engagement. Together, these results position reactive metallo‐scaffolds (***r*‐mS**) as a versatile platform for proteome‐wide covalent ligand discovery and the rational development of next‐generation metallodrugs.

## Graphical abstract

**Re‐envisioning the Ligandable Proteome**: Reactive Metallo‐Scaffolds (***r*‐mS**) combine non‐covalent protein engagement by a metallo‐scaffold with covalent cysteine capture through a tethered chloroacetamide warhead. Chemoproteomic profiling reveals unique enzyme engagement including ligases, kinases and methyltransferases, highlighting opportunities for expanding the druggable proteome and guiding the design of next‐generation metal‐based inhibitors and degraders.

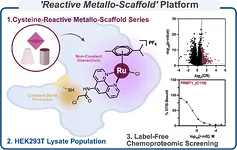

## 1 Introduction

Metal complexes lie at the heart of bioinorganic chemistry, underpinning essential biological processes and offering synthetic opportunities for diagnostics and therapy [1, 2, 3, 4, 5]. The discovery of cisplatin in 1965 established metal complexes as a cornerstone of cancer therapy. Since then, metallodrugs have emerged as compelling candidates for diverse therapeutic applications [4, 6, 7]. Their unique modular coordination environments, diverse geometries and physicochemical properties, combined with the high tunability afforded by ligand design, enable interactions with biomolecular targets that are difficult to achieve with purely organic molecules [4, 8, 9, 10]. These features also give rise to distinctive mechanisms of action, such as redox activity, photoactivation, radiochemistry and catalytic generation of reactive species [11, 12, 13, 14].

With a long‐standing presence in the clinic, metal complexes now extend beyond their established applications to remain indispensable in oncology and diagnostic imaging. In recent years, attention has turned to Group 8 metal complexes, with several advancing through clinical development. Among them, TLD‐1433, a ruthenium‐based photosensitiser in phase II clinical trials, is used in photodynamic therapy and induces cancer cell death through light‐activated generation of reactive oxygen species (ROS) [2, 15, 16]. BOLD‐100, another Ru complex, displays a unique mechanism of action centred around (1) altering the unfolded protein response (UPR) through selective GRP78 inhibition; and (2) the induction of ROS, which causes DNA damage and cell cycle arrest [17, 18, 19, 20]. Ferrocifen combines the redox activity of the iron complex ferrocene with tamoxifen‐like estrogen receptor modulation to promote oxidative stress and DNA damage [21, 22, 23]. These examples highlight the capacity of metal‐based therapeutics to engage both the genome and proteome. Several studies have begun to investigate their interactions with the proteome, revealing valuable, but fragmentary insights into target engagement in cell and lysate [24, 25]. Yet, unlike their well‐defined genomic targets, the protein interaction networks of clinically relevant complexes remain largely uncharacterised. Mapping these interactions will provide insight into mechanism of action and guide the rational design of next‐generation metallodrugs [26]. Pioneering work by Meggers and colleagues has shown that ruthenium complexes can mimic the kinase inhibitor staurosporine, illustrating how an inert metal centre can organise ligand presentation and molecular shape to enable precise protein recognition [27, 28, 29, 30, 31]. Building on these insights, we aim to systematically map how metal complexes interact with the human proteome, providing a foundation for rational metallodrug design and the discovery of novel therapeutic targets.

Chemoproteomics has emerged as a powerful and unbiased strategy to define how small molecules engage proteins across the proteome. Recently, the combination of covalent fragments and chemoproteomics has enabled rapid and robust mapping of ligandable sites across the proteome [32, 33, 34, 35, 36, 37, 38]. Covalent kinase inhibitors of EGFR (e.g., afatinib) and BTK (e.g., ibrutinib) have achieved notable clinical success in oncology [39, 40]. Although proteomic studies have begun to shed light on protein targets of metal complexes, the nature of these interactions can vary widely. In some reported cases, protein engagement arises from coordination of amino acid side chains—particularly cysteine thiolates—to the metal centre, or from redox‐mediated processes [9, 10, 41]. We reasoned that combining the unique reactivity of metal complexes with the cysteine‐targeting capabilities of covalent fragments would offer a powerful strategy to reveal the protein targets of clinically relevant metal complex scaffolds. This approach will enable elucidation of the complexes’ biological roles by disentangling their proteome‐wide interaction profiles, providing actionable insights for rational metallodrug design.

Herein, we report the development of reactive metallo‐scaffolds (***r*‐mS**) designed to interrogate metal complex‐protein interactions on a proteome‐wide scale. Each complex incorporates a tethered electrophilic chloroacetamide warhead into a diverse metallo‐scaffold, thereby combining a supramolecular and non‐covalent protein‐interaction component with a covalent cysteine‐labelling aspect. Importantly, ***r*‐mS** are distinct from classical metal‐thiol coordination complexes: in this study, cysteine labelling occurs through the chloroacetamide warhead, while the metal‐containing scaffold modulates molecular recognition, orientation, electronics, net charge and proteome‐wide engagement. The ***r*‐mSs** were profiled in cell lysate, followed by chemoproteomic analysis to identify liganded cysteines across the proteome. By tuning metal identity and ligand architecture, we examine how scaffold properties, including three‐dimensional structure, net charge and electronic features, dictate proteome‐wide reactivity and selectivity. This platform provides a generalisable tool for mapping metal complex‐protein interactions in cells, enabling the discovery of novel therapeutic targets and the optimisation of metallodrugs with tailored proteome selectivity.

## 2 Results and Discussion

### 2.1 ***r*‐mS** Selection and Reactivity

#### 2.1.1 Preparation of ***r*‐mS** for Chemoproteomic Screening

**FIGURE 1:**
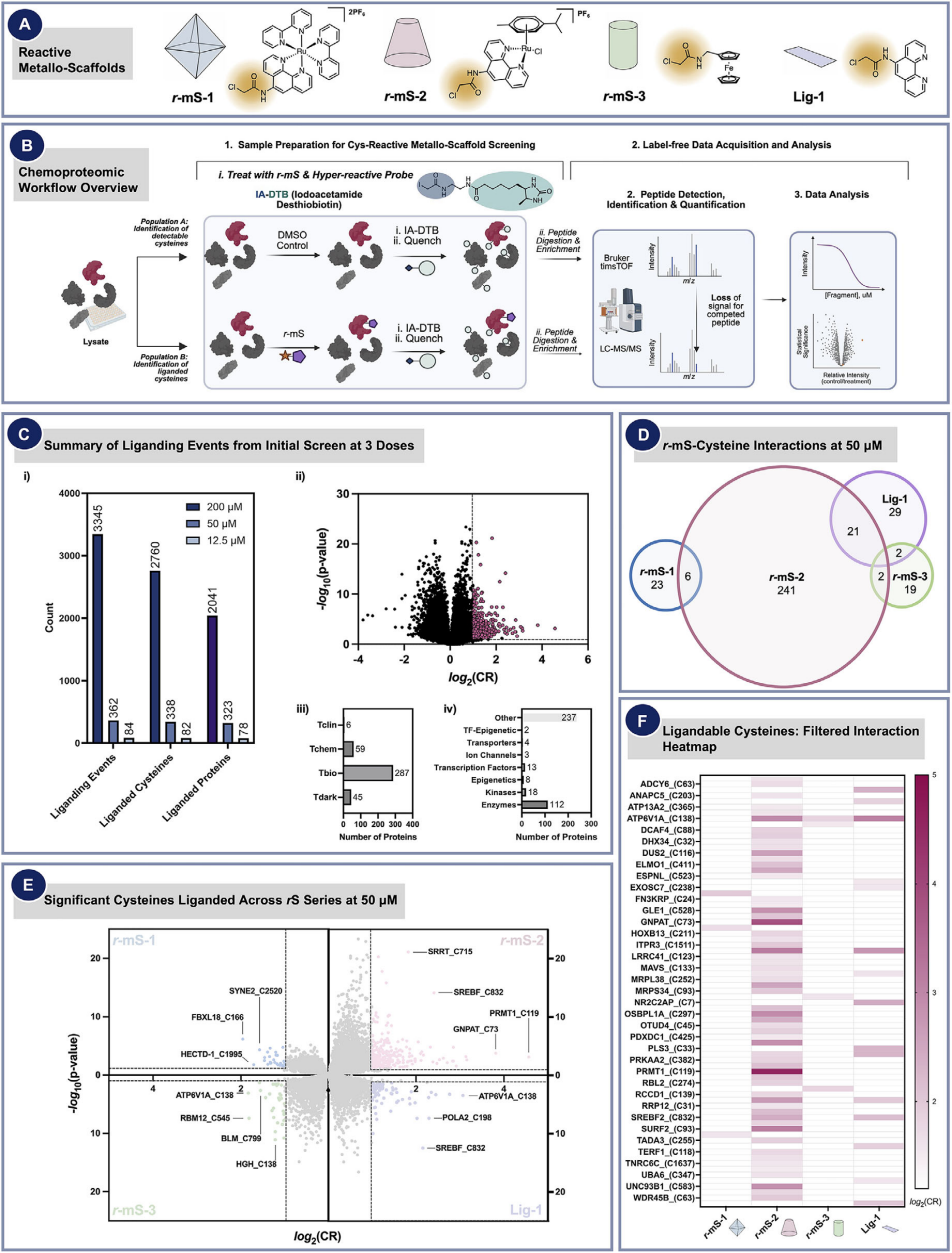
Reactive metallo‐scaffold screen performed in HEK293T lysate using HT‐LFQ chemoproteomics. (A) Structural and geometrical representations of the reactive metallo‐scaffolds (***r*‐mS**) evaluated in this study. (B) Overview of the label‐free sample preparation workflow and data acquisition strategy. The approach described in Stage 1 leverages a hyper‐reactive IA‐DTB probe to detect cysteine‐containing peptides from HEK293T lysates. Mass spectrometry analysis, detailed in Stage 2, was conducted using an Evosep One coupled to a Bruker timsTOF, with peptide identification and quantification performed via Spectronaut. Peptides covalently engaged by a ***r*‐mS** exhibit decreased intensity in compound‐treated samples compared to controls. (C) (i) Summary of liganding events, liganded cysteines and liganded proteins over 3 concentrations (200, 50, and 12.5 µM) from initial 3‐point concentration‐response proteomic screen of ***r*‐mS‐1**, ***r*‐mS‐2**, ***r*‐mS‐3**, and **Lig‐1**. (ii) Volcano plot summarising the experimental dataset, where each point represents an interaction between a cysteine‐containing peptide and a ***r*‐mS** (50 µM) in HEK293T lysate. Statistically significant liganding events, indicated by a strong competition ratio (CR) with IA‐DTB (log_2_(CR) ≥ 1.0 and ‐log_10_(*p‐*value) ≥ 1.3), are marked by dashed lines. (iii‐iv) Distribution of liganded proteins across ‘Illuminating the Druggable Genome’ protein families (iii) and target development categories (iv). (D) Four‐way Venn diagram depicting the overlap of liganding events identified across all tested compounds. Colours correspond to panels **A** and **E**. (E) Quadrant volcano plots highlighting significant liganding interactions for each ***r*‐mS** and the organic benchmark control **Lig‐1**. (F) Heatmap representing all detected interactions in HEK293T lysate at 50 µM, including only cysteine residues liganded by at least one compound with log_2_(CR) ≥ 1.0 and ‐log_10_(*p‐*value) ≥ 1.3.

We designed a series of ***r*‐mS** inspired by clinically or mechanistically studied metal complexes with diverse three‐dimensional structures and electronic properties [15, 20, 42]. We synthesised three geometrically‐distinct complexes incorporating: octahedral ruthenium (***r*‐mS‐1**), pseudo/distorted‐octahedral ruthenium (***r*‐mS‐2**), and the ferrocene‐based ***r*‐mS‐3** compound (Figure 1A and Scheme S1). Each incorporated an electrophilic chloroacetamide handle as the reactive moiety via the ligand. The chloroacetamide handle was selected for its well‐characterised reactivity with cysteine residues, synthetic accessibility, balanced electrophilicity, and established utility in covalent fragment‐based drug discovery [43, 44, 45]. Calculation of topological properties of candidate metal complexes using Principal Moment of Inertia (PMI) analysis confirmed that the selected scaffolds occupy regions of chemical space associated with high three‐dimensionality, with normalised PMI ratios of 0.72:0.86 (***r*‐mS‐1**), 0.67:0.97 (***r*‐mS‐2**) and 0.48:1.00 (***r*‐mS‐3**), each substantially displaced from rod‐ (1.0, 0.0) or disc‐like (0.5, 0.5) extremes (Figure S32). The synthesis of ***r*‐mS‐1** and ***r*‐mS‐2** began with the preparation of the corresponding chloroacetamide ligand (**Lig‐1**), followed by complexation to the ruthenium centre via *cis*‐dichlorobis(2,2’‐bipyridyl)ruthenium(II) (***r*‐mS‐1**) or [Ru(*p‐*cymene)Cl_2_]_2_ for ***r*‐mS‐2**. For ***r*‐mS‐3**, the reactive handle was introduced in the final step via an amide coupling with aminomethylferrocene [46, 47]. Stability tests confirmed that ***r*‐mS‐1** to ***r*‐mS‐3** remained intact in DMSO at −20° C for over two months, aligning with prior reports of chloroacetamide fragment stability in solution (Figure S36–S38) [32]. While ***r*‐mS‐1** and ***r*‐mS‐3** have been previously reported (***r*‐mS‐1** as a fluorescent DNA probe, and ***r*‐mS‐3** as a synthetic intermediate) to our knowledge, ***r*‐mS‐2** is reported here for the first time [48, 49, 50]. **Lig‐1** was employed throughout the study as an organic ligand control, enabling direct comparison with the ***r*‐mS** series to highlight the influence of metal complex structural diversity on protein binding.

#### 2.1.2 Assessment of Reactivity and Stability of ***r*‐mS**: QC Experiments

Initial efforts focused on ensuring that the ***r*‐mS** series were stable under standard chemoproteomics screening conditions. The stability of each ***r*‐mS** (***r*‐mS‐1**, ***r*‐mS‐2** and ***r*‐mS‐3**) was evaluated in radioimmunoprecipitation assay (RIPA) lysis buffer to mirror standard chemo proteomics conditions and was monitored over time by UPLC‐MS (Figures S33–S35). Reactive metallo‐scaffolds ***r*‐mS‐1**, ***r*‐mS‐2** and ***r*‐mS‐3** exhibited good stability over 1 h, this incubation time was therefore employed for the chemoproteomics profiling (Figures S33–S35). We acknowledge that aquation of ***r*‐mS‐2** is possible under aqueous conditions. However, given that the chemoproteomics assays are conducted in RIPA buffer containing excess sodium chloride (150 mM), extensive ligand exchange is suppressed, consistent with prior reports of suppressed Ru–S reactivity in chloride‐containing media) [51]. Correspondingly, UPLC–MS analysis (Figure S34) showed features consistent with the expected lability of the monodentate chlorido ligand in ***r*‐mS‐2**, including apparent chlorido ligand loss and formation of related ***r*‐mS‐2**‐derived species under the analytical conditions. While the high NaCl concentration of RIPA buffer may favour retention or re‐formation of the chlorido species, these data indicate that the chlorido ligand lability should not be excluded as a contributor to the behaviour of ***r*‐mS‐2** [51].

We evaluated the reactivity of ***r*‐mS‐2** with a panel of amino acids encompassing diverse chemical properties (*L*‐arginine, *L*‐histidine, *L‐*aspartic acid, *L*‐asparagine, *L*‐cysteine, *L*‐glutamic acid, *L*‐methionine and *L*‐lysine). ***r*‐mS‐2** was incubated with each amino acid in RIPA buffer (room temperature, pH 8.0), and the rate of ligation was assessed over time by UPLC‐MS. (Figure S40–S42). From this data, ***r*‐mS‐2** exhibited the highest reactivity towards *L*‐cysteine as evidenced by both depletion of the starting material and formation of the corresponding ***r*‐mS‐2**‐cysteine‐associated species. In contrast, no significant reactivity was observed with the other amino acids. Having established the selective reactivity of ***r*‐mS‐2** with cysteine, we extended this analysis to the additional ***r*‐mS** scaffolds and **Lig‐1**. Each compound was incubated with *L*‐cysteine for 90 min, and the rate of reaction determined. All compounds showed a time‐dependent reaction with cysteine, from which half‐lives were determined (***r*‐mS‐1** = 4.65 h, ***r*‐mS‐2** = 4.03 h, ***r*‐mS‐3** >24 h**, Lig‐1** = 7.52 h, Figure S43).

### 2.2 Chemoproteomic Screening of *r*‐mS Series

#### 2.2.1 Cysteinome Profiling by IA‐DTB

To systematically map the covalent interaction partners of our ***r*‐mS** series (***r*‐mS‐1**, ***r*‐mS‐2** and ***r*‐mS‐3**) as well as the organic control **Lig‐1**, we employed a label‐free chemoproteomic platform. This approach is designed for proteome‐wide profiling of cysteine reactivity in lysates, and provides an efficient and robust method to identify protein interactions [52]. In this method, compounds are profiled in competition with a hyper‐reactive iodoacetamide‐desthiobiotin (IA‐DTB) probe, which is used to label and enrich free cysteines for quantification by MS‐proteomics (Figure 1B) [53]. Competitive chemoproteomic profiling using IA‐DTB has been widely applied to benchmark cysteine engagement by electrophilic ligands and covalent fragments across the proteome. In this framework, compounds are evaluated based on their ability to compete with IA‐DTB for cysteine modification, providing a well‐established reference for assessing cysteine reactivity and selectivity [35, 43, 53, 54, 55]. This strategy has been used extensively to identify ligandable cysteines and to prioritise covalent fragments for subsequent optimisation. Intensities of enriched peptides were compared between reactive scaffold (***r*‐mS**)‐treated and DMSO control samples to calculate a competition ratio (CR), reflecting the extent of ***r*‐mS** labelling at each cysteine [CR = Intensity_DMSO_ / Intensity*_r_*_‐mS_]. Liganded peptides were defined as those with statistically significant covalent labelling that surpassed at least 75% cysteine occupancy ((log_2_(CR) ≥ 1.0, ‐log_10_(*p‐*value) ≥ 1.3) [52].

#### 2.2.2 Identification of Protein Targets for Reactive Metallo‐Scaffolds in HEK293T Lysate

Given that these compounds had not previously been tested in a proteome‐wide context and their relative potencies were unknown, we assessed proteome engagement at three concentrations. Each metallo‐scaffold (***r*‐mS‐1**, ***r*‐mS‐2** and ***r*‐mS‐3**), along with the organic ligand (**Lig‐1**), was tested in HEK293T cell lysate at 200, 50, and 12.5 µM (*n* = 3) at pH 8.0 for 1 h (Figure 1C‐i). This enabled us to map the concentration‐reactivity landscape and identify a productive yet controlled engagement window, with 50 µM providing an optimal balance between breadth of engagement and selectivity (Figure 1C‐ii). At this concentration, we identified 362 unique liganding events involving 338 cysteines across 323 proteins. Liganded proteins were first categorised by target development level (Figure 1C‐iii): ‘Tclin’ (approved drug targets), ‘Tchem’ (targets with known small‐molecule ligands), ‘Tbio’ (well‐characterised biology, but lacking chemical tools), and ‘Tdark’ (limited functional data) [56]. Notably, 84% of liganded proteins fell into the Tbio or Tdark categories, pointing to a substantial opportunity to develop ligands for previously untargeted proteins. Liganded targets spanned multiple protein superfamilies and functions, including 112 enzymes, 18 kinases, 13 transcription factors, 8 epigenetic regulators, as well as helicases, polymerases, proteases, E3 ligases, and receptors (Figure 1C‐iv). Comparative analysis of liganding across ***r*‐mS‐1**, ***r*‐mS‐2**, ***r*‐mS‐3** and **Lig‐1** revealed distinct proteome engagement patterns, with little overlap in engaged cysteines between scaffolds. ***r*‐mS‐2** showed the broadest profile, engaging 241 unique cysteine sites (Figure 1D). While most liganding events were scaffold‐specific, overlap was observed—most notably 21 shared sites between ***r*‐mS‐2** and **Lig‐1**. ***r*‐mS‐3** engaged 19 unique sites, sharing 2 each with ***r*‐mS‐2** and **Lig‐1**, while ***r*‐mS‐1** showed minimal overlap, sharing six sites with ***r*‐mS‐2**. These patterns reflect both scaffold‐specific selectivity and convergence at common hotspots. To identify the strongest cysteine interactions, we visualised the data using a four‐panel volcano plot (Figure 1E). ***r*‐mS‐2**, which engaged the largest number of cysteines, also resulted in the largest cysteine occupancies, with near complete competition of GNPAT_Cys73 (log_2_(CR) = 3.8, occupancy = 92%) and PRMT1_Cys119 (log_2_(CR) = 4.6, occupancy = 96%). In contrast, ***r*‐mS‐1** and ***r*‐mS‐3** exhibited narrower, more distinct profiles, suggesting scaffold‐dependent differences in electrophilicity or target accessibility. Notably, shared liganding events were observed at sites, such as ATP6V1A_Cys138 and SREBF1_Cys832, highlighting overlap in cysteine engagement across compounds. A composite heatmap, further highlighted the strength and selectivity of interactions across the three metallo‐scaffolds (Figure 1F), illustrating ***r*‐mS‐2**’s broad engagement relative to the more selective ***r*‐mS‐1** and ***r*‐mS‐3**.

**FIGURE 2:**
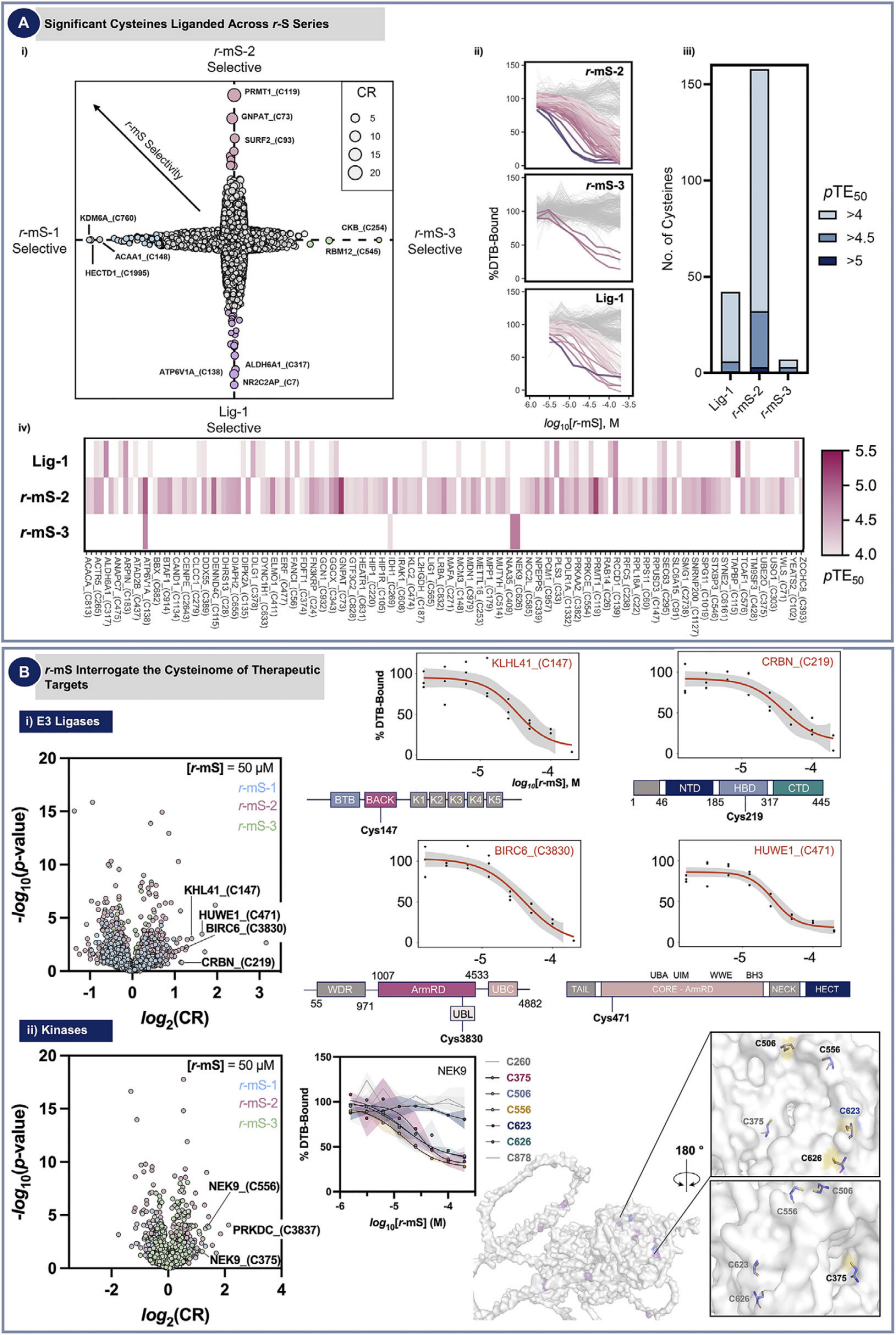
(A) (i) Four‐way plot highlighting the most selective cysteine engagements by ***r*‐mS**. Each point represents a cysteine interaction, with dot size corresponding to the magnitude of the competition ratio, reflecting the strength of engagement. (ii) 8‐point concentration‐response curves for ***r*‐mS‐2**, ***r*‐mS‐3** and **Lig‐1**. Curve colour corresponds to *p*TE_50_ values, ranging from pink to dark purple, with darker shades indicating higher levels of target engagement. (iii) Bar graph summarising the number of engaged cysteines for each compound, categorised by *p*TE_50_ thresholds (>4.0, >4.5, and >5.0) and colour‐matched accordingly. (iv) Heatmap representing cysteines liganded by **Lig‐1**, ***r*‐mS‐2** and ***r*‐mS‐3** with *p*TE_50_ values > 4.0. (B) Summary of cysteine liganding by ***r*‐mS** in proteins of therapeutic relevance. (i) E3 ligases: Volcano plot highlights selective cysteine engagement by ***r*‐mS‐1**, ***r*‐mS‐2** and ***r*‐mS‐3**, with ***r*‐mS‐2** responsible for liganding four E3 ligases: CRBN_Cys219, KLHL41_Cys147, HUWE1_Cys471, and BIRC6_Cys3830. Adjacent 8‐point concentration‐response curves display compound‐dependent engagement, with liganded cysteines annotated within each ligase's domain architecture. (ii) Kinases: Volcano plot shows selective cysteine reactivity in NEK9_Cys375, NEK9_Cys556 and PRKDC_Cys3837 following treatment with ***r*‐mS‐1**, ***r*‐mS‐2** and ***r*‐mS‐3**. Concentration‐response profiling of NEK9 revealed multiple cysteines—NEK9_Cys375, NEK9_Cys506, NEK9_Cys556 and NEK9_Cys626—exhibiting concentration‐dependent engagement by ***r*‐mS‐3**, while others (NEK9_Cys260, NEK9_Cys878) remained unmodified. Structural mapping of NEK9 (based on the AlphaFold model) indicates that the responsive cysteines cluster around a defined surface pocket, suggesting that the ***r*‐mS** may sterically occlude several proximal residues through a single binding event.

To identify the most selective interactions, we plotted differential cysteine engagement for ***r*‐mS‐1**, ***r*‐mS‐2**, ***r*‐mS‐3** and **Lig‐1** (Figure 2A‐i). Each compound displayed a panel of highly selective cysteine targets, underscoring the influence of scaffold shape and electronics on recognition. Among these, Cys119 liganded in PRMT1 by ***r*‐mS‐2** was the strongest and most scaffold‐specific interaction. To further assess potency, selectivity and validate hits from the initial screen, we chose to screen scaffolds ***r*‐mS‐2** and ***r*‐mS‐3** in HEK293T cell lysate via 8‐point concentration‐response (1.6‐200 µM, triplicate measurements, Figure 2A‐ii) along with **Lig‐1** as a control. These scaffolds were prioritised over ***r*‐mS‐1**, which yielded fewer compelling engagement patterns in the initial screen. Screening at multiple concentrations enabled curve fitting to calculate half‐maximal target engagement (TE_50_) values for ***r*‐mS‐2**, ***r*‐mS‐3** and **Lig‐1**, defined as the concentration at which 50% of a given cysteine site is bound. The resulting curves revealed clear, scaffold‐specific engagement profiles. ***r*‐mS‐2** liganded the greatest number of targets in a concentration‐dependent manner, recapitulating the observations from the 3‐point concentration experiments. To further interpret this dataset, we extracted *p*TE_50_ values (i.e., ‐log_10_(TE_50_) values) and applied filters based on curve quality and potency (Figure 2A‐iii) to determine the number of cysteines engaged above *p*TE_50_ thresholds of 4.0, 4.5 and 5.0 [52]. ***r*‐mS‐2** liganded 126 cysteines with 4 < *p*TE_50_ ≤4.5, 29 above 4.5, and 3 exceeding 5.0. In comparison, **Lig‐1** engaged 36 cysteines above *p*TE_50_ 4.0, 5 above 4.5, and just one above 5.0, while ***r*‐mS‐3** showed the narrowest profile with 4, 3 and 0 cysteines above each threshold, respectively. A heatmap of these filtered liganding events showed strong agreement with hits from the initial screen (Figure 2A‐iv). Together, these analyses provide a map of scaffold‐driven cysteine targeting, reinforcing that selective and potent covalent interactions are achievable with ***r*‐mS**.

To underscore the translational potential of our approach, we focused on protein families of high therapeutic relevance, including kinases and E3 ubiquitin ligases. The latter play central roles in cell signaling and have emerged as pivotal drivers of targeted protein degradation strategies [57]. At 50 µM, multiple ***r*‐mS** liganding events were identified across ligases involved in substrate recognition, signaling, and degradation pathways (Figure 2B‐i). Notably, ***r*‐mS‐2** engaged CRBN_Cys219 (*p*TE_50_ = 4.6 ± 0.09), KLHL41_Cys147 (*p*TE_50_ = 4.7 ± 0.08), BIRC6_Cys3830 (*p*TE_50_ = 4.7 ± 0.06), and HUWE1_Cys471 (*p*TE_50_ = 4.6 ± 0.05) (Figure 2B‐i, right). Mapping these engagement sites onto annotated E3 ligase domain architectures contextualised these interactions and highlighted potential functional consequences. CRBN, the E3 ligase most commonly recruited in proteolysis targeting chimera (PROTAC) development, was liganded at Cys219 ‐ a previously untargeted site within the Damage specific DNA binding protein1 (DDB1)—binding helical bundle (HB) domain—offering opportunities to modulate complex assembly at a novel, tractable residue which has been demonstrated to be an effector of protein ubiquitination [58, 59]. BIRC6, a target of therapeutic interest in oncology and immunology, was liganded at Cys3830 in the substrate‐recognition solenoid, suggesting a path to modulate client capture [60, 61, 62]. KLHL41, with >60‐fold higher expression in skeletal muscle than any other tissue, due to its super‐enhancer architecture, was liganded at Cys147 within the BACK domain, potentially modulating scaffold formation in a tissue‐selective ligase [63, 64, 65, 66]. HUWE1, an emerging ligase of interest, was liganded at Cys471 in the α‐solenoid ring scaffold, where engagement may allosterically influence substrate docking or access to the catalytic HECT domain [67]. These findings highlight the ability of ***r‐*mS‐2** to engage functionally relevant, noncatalytic cysteines in both well‐characterised and understudied E3 ligases. This demonstrates the potential of our method to inform the development of covalent probes for the exploration and modulation of protein degradation pathways.

We next examined kinase liganding events, where ***r*‐mS‐3** displayed more diverse reactivity across the kinome (Figure 2B‐ii). At 50 µM, multiple kinases were liganded across several families, with PRKDC (***r*‐mS‐2**), and NEK9 (***r*‐mS‐3**) showing the strongest overall engagement. Interestingly, multiple cysteine sites of NEK9 were competed by ***r*‐mS‐3**, with Cys375, Cys506, Cys556, and Cys626 displaying concentration‐dependent reduction. In contrast, (Cys260, Cys878) showed no change across concentrations, suggesting that the observed competition was not a by‐product of protein aggregation. Previously, electrophilic compounds have been reported to target Cys623 in NEK9 (blue annotation in the protein structure of Figure 2B‐ii) [68]. In our experiments, we likewise observed liganding of Cys623, alongside robust engagement of the adjacent Cys626. Structural analysis using the AlphaFold model of NEK9 (AF‐Q8TD19‐F1) revealed that these concentration‐responsive cysteines cluster spatially around the rim of a defined pocket, highlighting this region as a potential hotspot for covalent ligand binding. Given this arrangement, the observed engagement pattern is more consistent with ligand‐induced conformational changes than with simple steric occlusion, placing this example alongside our observations for Cys260 and Cys878. Such behaviour may reflect a broader capability of ***r*‐mS** to modulate cysteine reactivity through conformational effects.

We examined the structural characteristics of cysteines targeted by ***r*‐mS** and **Lig‐1** at 50 µM (Figure S44). For each cysteine, we computed various structural descriptors—including physiochemical properties, local neighbourhood features, and properties of the closest binding pocket [69]. We then compared the distributions of these features between **Lig‐1** and the reactive metallo‐scaffolds (combined and individually; Figure S44a). In general, ***r*‐mS** targeted cysteines that were more structurally ordered and buried, and were associated with a broader range of predicted *p*Ka values than those engaged by **Lig‐1**. Notably, the non‐neutral ***r*‐mS‐1** and ***r*‐mS‐2** scaffolds interacted significantly more with pockets bearing a net charge, highlighting a greater role for electrostatic interactions (Figure S44b). Although pocket volume was not a distinguishing factor overall, cysteines engaged by ***r*‐mS‐1** and ***r*‐mS‐2** tended to reside in smaller pockets. Interestingly, ***r*‐mS‐3** exhibited the most pronounced differences relative to **Lig‐1**, possibly because it lacks **Lig‐1** as a ligand. It is important to interpret these findings with caution due to limited sample sizes and reliance on predicted rather than experimentally determined structures, which may introduce noise, especially for cysteines in disordered regions or protein‐protein interfaces that appear solvent exposed in monomeric models [70, 71, 72].

Together, these findings demonstrate the ability of ***r*‐mS** to access functionally relevant, noncatalytic cysteines across challenging target classes. This capacity for site‐selective engagement offers a promising foundation for exploring and modulating previously underexploited regions of the proteome.

### 2.3 Second‐Generation Scaffolds of ***r*‐mS‐2**

**FIGURE 3:**
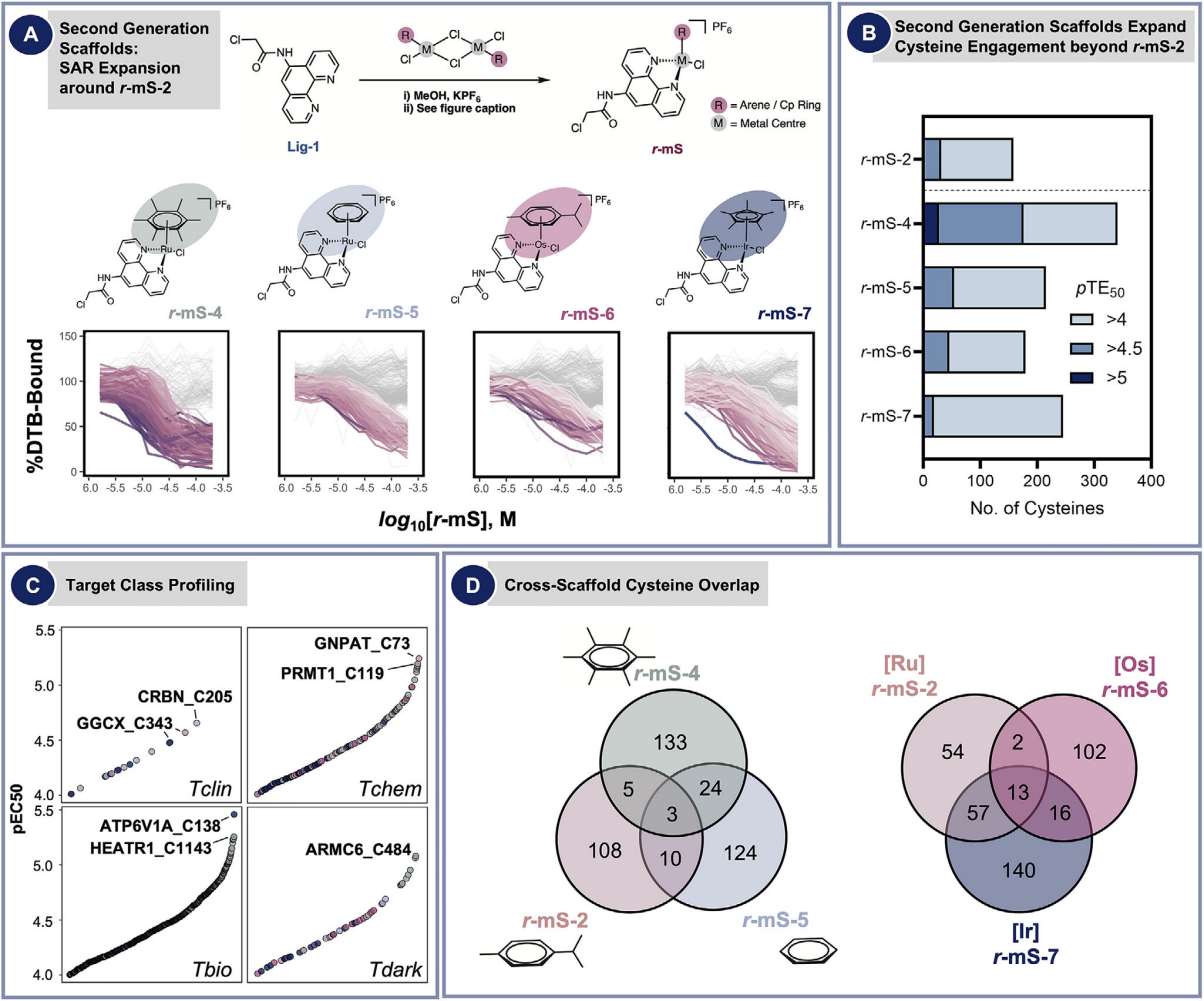
Summary of liganding events pertaining to ***r*‐mS‐2** derivatives. (A) Structural expansion around ***r*‐mS‐2** led to the synthesis of second‐generation analogues (***r*‐mS‐4**, ***r*‐mS**‐**5**, ***r*‐mS**‐**6** and ***r‐*mS‐7**), each featuring modifications to the ligand core, arene, or metal centre. General synthetic procedure outlined in the scheme. Briefly, ***r*‐mS‐4**, ***r*‐mS‐5** and ***r*‐mS‐6** were prepared by combining the corresponding Ru or Os dimer with **Lig‐1** and KPF_6_ in methanol, followed by sonication for 15 min and filtration to afford the respective metallo‐scaffolds. For ***r*‐mS‐7**, the analogous Ir dimer, **Lig‐1**, and KPF_6_ were stirred in methanol overnight and then filtered to yield the corresponding complex. Concentration‐response curves for each compound show cysteine engagement profiles in HEK293T lysate, with darker shades indicating higher levels of target engagement. (B) Bar chart quantifying total cysteine engagements for each compound, binned by *p*TE_50_ threshold (≥ 4.0, 4.5 or 5.0). Second‐generation compounds expand cysteine coverage beyond ***r*‐mS‐2**, with ***r*‐mS‐4** displaying the broadest engagement. (C) Chemoproteomic mapping reveals target class distribution: cysteine‐labelled proteins annotated by Tclin, Tdark, Tbio and Tchem classifications. (D) Cross‐scaffold overlap of liganded cysteines for each ***r*‐mS**, filtered to include only sites displaying a concentration‐response relationship. Left: Venn diagram showing overlap of cysteine engagements across ***r*‐mS‐2**, ***r*‐mS‐4** and ***r*‐mS‐6**, highlighting differences in proteome coverage between various arene hats. Right: Overlap analysis shows scaffold‐specific and shared reactivity, highlighting differences in proteome coverage between metal centre [Ru] (e.g., ***r*‐mS‐2**) and [Os] (e.g., ***r*‐mS‐6**).

With ***r*‐mS‐2** emerging as the most productive scaffold, we designed a focused panel of second‐generation scaffolds (***r*‐mS‐4**, ***r*‐mS‐5**, ***r*‐mS‐6** and ***r*‐mS‐7**) to explore how subtle modifications to coordination environment affect proteome‐wide interactions and assess structure‐activity relationship(s) (SAR). The second‐generation scaffolds varied in metal centre and arene ligand (Figure 3A, top) and were synthesised via established methods: **Lig‐1** was reacted with the appropriate metal dimer in methanol to yield piano‐stool complexes with distinct topologies: ***r*‐mS‐4** (Ru‐hexamethylbenzene), ***r*‐mS‐5** (Ru‐benzene), ***r*‐mS‐6** (Os‐*p‐*cymene) and ***r*‐mS‐7** (Ir‐Cp*) (Figure 3A, middle, Scheme S1). To assess how these changes impact reactivity and selectivity, each compound was screened across an 8‐point concentration‐response series in HEK293T lysate. To compare potency across the second‐generation series, we quantified the number of cysteines engaged above *p*TE_50_ thresholds of 4.0, 4.5 and 5.0 (Figure 3B). All four analogues exhibited broad and generally productive proteome‐wide engagement, with ***r*‐mS‐4** standing out for both the number of cysteines targeted and the potency of liganding events. ***r*‐mS‐7** demonstrated a comparable number of liganded sites, albeit with less potency relative to ***r*‐mS‐4**. These results suggest that both metal identity, arene electronics and hydrophobicity play key roles in shaping cysteine reactivity and overall scaffold performance. To further explore the breadth of proteome reactivity, we examined the target class distribution of liganded proteins, and the degree of cysteine overlap across the second‐generation scaffolds and ***r*‐mS‐2** (Figure 3C, D). All scaffolds were found to engage proteins across the four classes of target development, and the majority of proteins were found to be liganded specifically by one scaffold. To dissect the influence of scaffold composition on cysteine targeting, we compared subsets of scaffolds in two focused Venn analyses (Figure 3D). In the first comparison (left), scaffolds with varying arene caps—hexamethylbenzene (***r*‐mS‐4**), benzene (***r*‐mS‐5**) and *p‐*cymene (***r*‐mS‐2**)—comprising a conserved Ru(II) centre—were compared. The probes exhibited a remarkably small degree of overlap of the cysteines engaged by all three, indicating that the steric and/or electronics of the arene caps plays a key role in the protein recognition. Here, ***r*‐mS‐4** showed the greatest number of unique engagements, followed closely by ***r*‐mS‐5**, with notable overlap between them, while ***r*‐mS‐2** showed comparatively little overlap with either. In the second comparison (right), varying the metal centre (Ru, Os, Ir) revealed a clear trend: Ir > Os > Ru with regards to total cysteine engagement. Interestingly, there was a higher degree of overlap of the proteins engaged by each ***r*‐mS**, though the majority of interactions were still unique to a single scaffold. Although the metal is not the site of covalent bond formation in these interactions, it exerts a pronounced influence on the stereo‐electronic features of the complex and the electrophilicity of the coordinated chloroacetamide. The higher number of liganding events of the Ir(III) complex may arise from the greater Lewis acidity and stronger electron‐withdrawing nature of the Cp*‐Ir(III) portion, which inductively increases the polarisation of the C─Cl bond. In contrast, Ru(II) arene complexes exhibit lower electrophilic activation of the warhead, potentially due to weaker metal‐to‐ligand inductive effects and greater resonance stabilisation of the amide. Steric factors may also contribute: The Cp* ligand on Ir, while bulky, may promote an orientation that facilitates nucleophilic attack, whereas the more planar *p‐*cymene ligands on Ru and Os may impose geometric constraints that limit warhead accessibility. The apparent trends likely reflect a composite of effects from differences in lipophilicity, electrophile activation, and steric approachability across the series [73].

### 2.4 ***r*‐mS‐2** and PRMT1

#### 2.4.1 Selective Engagement and Functional Activity of ***r*‐mS‐2** Against PRMT1

**FIGURE 4:**
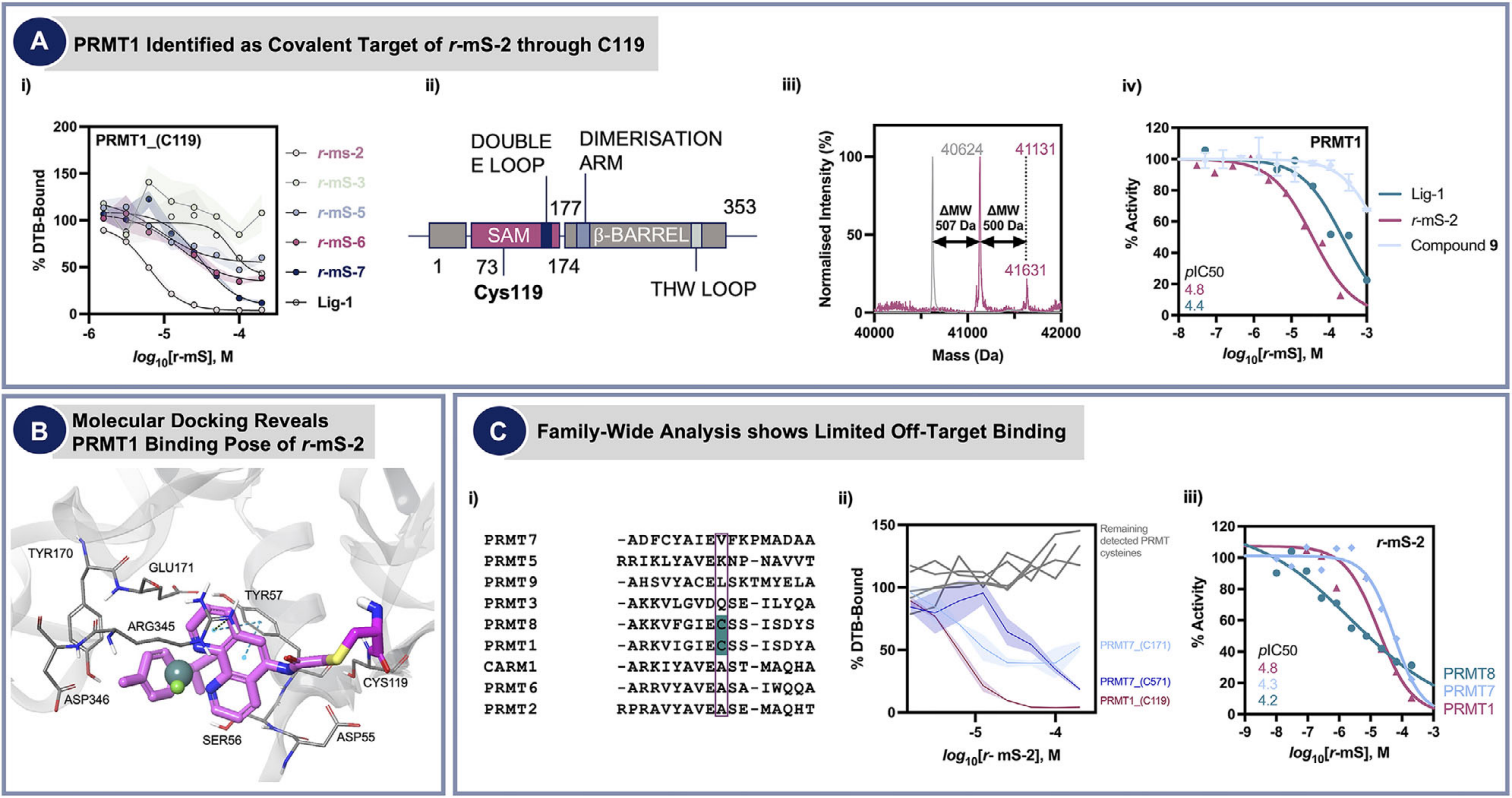
PRMT1 is a selective covalent target of ***r*‐mS‐2** through Cys119. (A)(i) 8‐point concentration response curves from chemoproteomic profiling show Cys119 engagement by ***r*‐mS**, with ***r*‐mS‐2** exhibiting the strongest most consistent binding. ***r*‐mS‐4** was excluded from this figure due to an anomalous response profile and poor concentration‐response fit; full data are provided in the Supporting Information (Figure S45) for transparency. (A)(ii) Sequence domain architecture of PRMT1, indicating location of Cys119 within the SAM‐binding domain. (A)(iii) IP‐LC‐MS spectrum confirming covalent modification of PRMT1 by ***r*‐mS‐2** (507 Da). A secondary peak at 500 Da lies outside the instrument's mass accuracy window and is therefore unlikely to represent an additional ***r*‐mS‐2** adduct. (A)(iv) In vitro HMT assay confirms functional inhibition of PRMT1 by ***r*‐mS‐2**. Dose‐response curves for ***r*‐mS‐2**, **Lig‐1**, and the non‐reactive metallo‐scaffold control (compound **9**) show ***r*‐mS‐2** exhibits the most potent inhibition, consistent with covalent engagement. (B) Molecular docking of ***r*‐mS‐2** with PRMT1 reveals a binding pose consistent with covalent modification. Key stabilising interactions include cation‐π stacking with Arg345 and π–π stacking with Tyr57, orienting the electrophilic moiety towards Cys119. (C)(i) Sequence alignment of PRMT isoforms centred on PRMT1_Cys119. The reactive site is not conserved across the family, with the exception of PRMT8, which shares a homologous cysteine (teal highlight). However, PRMT8 was not detected in the chemoproteomic dataset, likely due to low endogenous expression or limited MS detectability under the applied conditions. (C)(ii) Concentration‐response curves from chemoproteomic profiling of PRMT family members. PRMT1_Cys119 shows the strongest and most consistent engagement by ***r*‐mS‐2** across concentrations. Minor off‐target reactivity is observed at PRMT7_Cys571 and PRMT7_Cys171, while other isoforms show minimal to no detectable engagement. (C)(iii) In vitro HMT assay assessing functional inhibition of PRMT1, PRMT7 and PRMT8 by ***r*‐mS‐2**. Consistent with proteomic data, ***r*‐mS‐2** shows potent inhibition of PRMT1, with only minor off‐target effects observed for PRMT7 and PRMT8.

Recognising potent engagement of PRMT1_Cys119 by ***r*‐mS‐2**, we undertook further studies to characterise this interaction. PRMT1 is the most active arginine methyltransferase in humans, catalysing asymmetric demethylation of arginine residues and regulating gene expression, RNA processing, DNA repair and signal transduction. Its activity is essential for cellular growth and differentiation, and dysregulation has been linked to cancer, cardiovascular disease and neurological disorders [74, 75, 76, 77, 78]. To reaffirm the PRMT1_Cys119 engagement observed for ***r*‐mS‐2** in the 3‐point screen, we referred back to the corresponding 8‐point concentration‐response dataset, which confirmed concentration‐dependent binding (*p*TE_50_ = 5.2 ± 0.01). Among the compounds tested, ***r*‐mS‐2** exhibited the most pronounced engagement of PRMT1. In contrast, other ***r*‐mS** analogues displayed either weaker engagement (***r*‐mS‐7**, ***r*‐mS‐6**, ***r*‐mS‐5**) or no engagement (***r*‐mS‐3**, **Lig‐1**, Figure 4A‐i). To better understand the structural context of this interaction, we examined the domain architecture of PRMT1 (Figure 4A‐ii). The liganded cysteine (Cys119) is located within the *S*‐adenosylmethionine (SAM) binding domain, a region critical for coordinating the methyl donor used in arginine methylation. Inhibitors aimed at modulating aberrant PRMT1 activity target this SAM‐binding domain. Among the most active PRMT1 ligands are MS023 (IC_50_ = 30 nM), DCLX069 (IC_50_ = 18 µM, highly selective), and the cell‐permeable inhibitors AMI‐1 (IC_50_ = 9 µM) and GSK3368715 (IC_50 _= 3 nM), with IC_50_ values reflecting the concentration at which half‐maximal inhibition is achieved. Notably, none of the reported ligands for PRMT1 are covalent binders [79, 80, 81, 82]. The ability of ***r*‐mS‐2** to selectively and covalently engage Cys119 within this functional domain highlights its potential as a scaffold for developing targeted covalent inhibitors of PRMT1 and related methyltransferases. Cys119 has garnered particular interest in recent literature for its role in modulating PRMT1 activity, particularly under oxidative and nitrosative stress conditions. Modifications at this site, including *S*‐nitrosylation and oxidation to sulfinic or sulfonic acid have been reported to impair enzymatic function [83].

To confirm target engagement, we assessed the covalent interaction between ***r*‐mS‐2** and PRMT1 using recombinant protein. Analysis of PRMT1 by intact protein mass spectrometry (IP‐LC‐MS) following incubation with ***r*‐mS‐2** for 1 h confirmed single covalent labelling of PRMT1 (Figures 4A‐iii, S47), supporting direct covalent modification of the protein. To confirm site of modification, recombinant PRMT1 was subjected to tryptic digestion and was analysed by peptide‐level LC‐MS/MS, revealing exclusive liganding at Cys119 (100% of observed modifications; Figure S48). We next employed a recombinant histone methyltransferase (HMT) assay to assess compound‐dependent modulation of PRMT1 enzymatic activity (Figure 4A‐iv). For comparison, *S*‐(5'‐Adenosyl)‐*L*‐homocysteine (SAH), a known PRMT1 methylation product, was tested under similar conditions. Following a 1 h incubation, ***r*‐mS‐2** exhibited moderate potency against PRMT1, with an IC_50_ of 15 µM. In contrast, reactive scaffold benchmark **Lig‐1** and a non‐covalent analogue of ***r*‐mS‐2** (compound **9**, Table S2) both showed weaker inhibition, highlighting the importance of both the molecular topography and the covalent binding.

#### 2.4.2 Molecular Recognition

To illustrate a plausible pre‐reactive orientation of ***r*‐mS‐2** in PRMT1, we docked into a static receptor model using Cys119 as the anchoring site. Initial non‐covalent docking (MetalDock) generated a pre‐reactive pose that was refined with AutoDock's covalent protocol [84, 85]. The model suggests stabilising interactions for the cationic headgroup (cation–π with Arg345; π–π with Tyr57) that could guide the scaffold into a reactive geometry (Figure 4B). In this pose, the arene substituent extends towards solvent and does not engage a well‐defined subsite, consistent with limited specific contribution from that moiety. We use this pose as an illustrative structural framework to orient discussion of site features, while interpreting relative potency and ‘arene‐site engagement’ across ***r*‐mS‐2**/***r*‐mS‐4**/***r*‐mS‐5** in the broader context of effects not captured by a single rigid‐receptor snapshot. Accordingly, we treat this pose as a qualitative guide, recognising that differences in engagement and potency amongst the close analogues can arise from factors not captured by a single rigid‐receptor docking model.

#### 2.4.3 Reactivity of ***r*‐mS‐2** Towards Other Methyltransferases

PRMTs are categorised into three classes based on their methylation outputs: Type I PRMTs catalyse asymmetric demethylation (ADMA, including PRMT1), Type II PRMTs catalyse symmetric demethylation (SDMA), and Type III PRMTs catalyse monomethylation (MMA) [86]. The SAM‐binding domain is conserved among PRMTs and many PRMT1 inhibitors show broad‐spectrum activity, especially within the Type I family. A sequence alignment of PRMTs highlighted low conservation of Cys119, with only PRMT8 (Type I) containing an analogous cysteine, suggesting Cys119 targeting covalent inhibitors could confer exquisite selectivity (Figure 4C‐i). To evaluate specificity, we assessed the reactivity of ***r*‐mS‐2** across the PRMT family (Figure 4C‐ii). Initially, we focused on identifying PRMT cysteines that exhibited concentration‐dependent competition of IA‐DTB by ***r*‐mS‐2** within cell lysates. Five cysteines across PRMT3, PRMT5, PRMT6, PRMT7 and PRMT9 were robustly quantified, of which only PRMT7_Cys171 was competed in a concentration‐dependent manner by ***r*‐mS‐2** (*p*TE_50_ = 5.2 ± 0.09). No peptides corresponding to PRMT8 were detected in the IA‐DTB dataset, likely reflecting low endogenous expression rather than a lack of compound engagement. PRMT7_Cys171 is located in the SAM‐binding domain of PRMT7, suggesting a conserved binding mechanism of ***r*‐mS‐2** between PRMT7 and PRMT1. The inhibitory activity of ***r*‐mS‐2** was assessed for the two potential PRMT off‐targets identified by chemoproteomics (PRMT7) and in silico sequence alignment (PRMT8) yielding inhibition of these proteins with comparable potency: PRMT7 *p*IC_50_ = 4.3, PRMT8 *p*IC_50_ = 4.2, PRMT1 *p*IC_50_ = 4.8 (Figure 4C‐iii). These results demonstrate that ***r*‐mS‐2** may serve as a promising starting point for the development of PRMT1/PRMT7/PRMT8‐selective chemical probes that avoid pan‐PRMT activity. Further studies are, however, warranted to fully elucidate selectivity across the broader PRMT family and improve on‐target potency.

### 2.5 Conclusions and Outlook

This study establishes reactive metallo‐scaffolds (***r*‐mS**) as a versatile platform for covalent ligand discovery and proteome‐wide target engagement. Through chemoproteomic profiling, we demonstrate that ***r*‐mS**—particularly ***r*‐mS‐2**—covalently engages functionally relevant cysteine residues across diverse protein classes, including kinases and E3 ligases. The identification and biochemical validation of PRMT1_Cys119 binding, leading to functional inhibition, highlights the potential of ***r*‐mS** to target enzymes via covalent interactions. Scaffold reactivity was found to be tunable through the variation of metal centre and ligand architecture, providing a roadmap for further optimisation. Importantly, ***r*‐mS‐2** selectively engaged PRMT1 over closely related isoforms and uniquely targeted proteins, such as BIRC6 and HUWE1, which are not liganded in existing chemoproteomic scout or reactive fragment libraries. This underscores their ability to access structurally distinct, underexplored pockets within the proteome. These findings demonstrate the promise of metallo‐based covalent fragments for expanding the druggable proteome and developing next‐generation inhibitors and degraders, offering a blueprint for the integration of metal coordination chemistry into modern covalent drug discovery.

### 2.6 Figure Preparation

Figure S1 was created using image templates from BioRender.com under licensing from the Francis Crick Institute. All graphs and charts were plotted using Graphpad Prism 10.0 or RStudio (v 4.3.3). Chemo proteomics data was filtered using appropriate hit criteria and visualised in Spotfire, before being replotted in Graphpad Prism 10.0. or RStudio (v 4.3.3).

## Author Contributions

Syntheses of all precursors and scaffolds and all QC experiments were performed by **Jessica E. Waters**. **Jessica E. Waters** and **George S. Biggs**, carried out proteomics experiments and initial data analysis; **Harry Wilders** assisted with proteomics‐related data analysis interpretation, figure making, proof reading, and editing; **Emma E. Cawood** carried out all downstream data analysis, **Jessica E. Waters** carried out data visualizations and figure design; **Jonathan Bailey** cloned, expressed and purified PRMT1 and co‐performed PRMT1 mass spectrometry sample preparation; **Mika Kintzel** assisted with Python scripts and additional QC incubation experiments; intact mass spectrometry experiments were run by **Sarah Maslen**; **Yew Mun Yip** performed docking calculations and performed analysis for Figure 4 using PyMOL, AutoDock, MetalDock, and Maestro software. **Sarah Maslen** performed all mass spectrometry experiments on incubations between ***r*‐mS‐2** and PRMT1. **Ioannis G. Riziotis** carried out all structural feature extraction and statistical modelling. **Mika Kintzel** assessed reactivity of ***r*‐mS**, **Lig‐1** and compound **9** with cysteine. **Thomas W. Rees** assisted proof reading and generation of final NMR spectra and assignments. **Jeannine Hess** obtained funding and supervised project; **Jessica E. Waters** and **George S. Biggs** conceptualised the project, with assistance from **Jacob Bush** and **Jeannine Hess**. **Jessica E. Waters** wrote the paper with contributions from all authors. PRMT‐specific HMT IC_50_ experiments were run by Reaction Biology Ltd.

## Conflicts of Interest

The authors declare no conflicts of interest.

## Supporting information




**Supporting File**: anie73354‐sup‐0001‐SuppMat.docx.

## Data Availability

The raw mass spectrometry proteomics files and database search results have been deposited at the ProteomeXchange Consortium [87], via the PRIDE partner repository with data set identifiers PXD068320 [88].

## References

[1] K. Peng , Y. Zheng , W. Xia , and Z.‐W. Mao , “Organometallic Anti‐Tumor Agents: Targeting From Biomolecules to Dynamic Bioprocesses,” Chemical Society Reviews 52 (2023): 2790–2832, 10.1039/D2CS00757F. 37014670

[2] S. Monro , K. L. Colón , H. Yin , et al., “Transition Metal Complexes and Photodynamic Therapy From a Tumor‐Centered Approach: Challenges, Opportunities, and Highlights From the Development of TLD1433,” Chemical Reviews 119 (2019): 797–828, 10.1021/acs.chemrev.8b00211. 30295467 PMC6453754

[3] G. Gasser , I. Ott , and N. Metzler‐Nolte , “Organometallic Anticancer Compounds,” Journal of Medicinal Chemistry 54 (2011): 3–25, 10.1021/jm100020w. 21077686 PMC3018145

[4] E. J. Anthony , E. M. Bolitho , H. E. Bridgewater , et al., “Metallodrugs Are Unique: Opportunities and Challenges of Discovery and Development,” Chemical Science 11 (2020): 12888–12917, 10.1039/D0SC04082G. 34123239 PMC8163330

[5] J. E. Waters , L. Stevens‐Cullinane , L. Siebenmann , and J. Hess , “Recent Advances in the Development of Metal Complexes as Antibacterial Agents With Metal‐Specific Modes of Action,” Current Opinion in Microbiology 75 (2023): 102347, 10.1016/j.mib.2023.102347. 37467616

[6] S. Rottenberg , C. Disler , and P. Perego , “The Rediscovery of Platinum‐Based Cancer Therapy,” Nature Reviews Cancer 21 (2021): 37–50, 10.1038/s41568-020-00308-y. 33128031

[7] B. Rosenberg , L. V. Camp , and T. Krigas , “Inhibition of Cell Division in *Escherichia coli* by Electrolysis Products From a Platinum Electrode,” Nature 205 (1965): 698–699, 10.1038/205698a0. 14287410

[8] A. Casini and A. Pöthig , “Metals in Cancer Research: Beyond Platinum Metallodrugs,” ACS Central Science 10 (2024): 242–250, 10.1021/acscentsci.3c01340. 38435529 PMC10906246

[9] M. Dijkstra , H. Schueffl , B. Adamova , et al., “Exploring the Structure–Activity Relationships of Albumin‐Targeted Picoplatin‐Based Platinum(IV) Prodrugs,” Inorganic Chemistry 64 (2025): 2554–2566, 10.1021/acs.inorgchem.4c05269. 39878587 PMC11815855

[10] B. Das and P. Gupta , “Multimetallic Transition Metal Complexes: Luminescent Probes for Biomolecule Sensing, Ion Detection, Imaging and Therapeutic Application,” Coordination Chemistry Reviews 504 (2024): 215656, 10.1016/j.ccr.2024.215656.

[11] S. Spreckelmeyer , S. R. Thomas , F. Schuderer , et al., “N‐Heterocyclic Carbenes as Ligands to 198Au(I)‐Radiolabeled Compounds: A New Platform for Radiopharmaceutical Design,” Journal of Medicinal Chemistry 68 (2025): 17516–17526, 10.1021/acs.jmedchem.5c01073. 40797268 PMC12406186

[12] E. Boros , P. J. Dyson , and G. Gasser , “Classification of Metal‐Based Drugs According to Their Mechanisms of Action,” Chemistry 6 (2020): 41–60, 10.1016/j.chempr.2019.10.013. PMC7451962 32864503

[13] E. Meggers , “Targeting Proteins With Metal Complexes,” Chemical Communications 45 (2009): 1001–1010, 10.1039/b813568a. 19225621

[14] L. Riccardi , V. Genna , and M. De Vivo , “Metal–Ligand Interactions in Drug Design,” Nature Reviews Chemistry 2 (2018): 100–112, 10.1038/s41570-018-0018-6.

[15] Theralase® Technologies Inc ., *A Phase II Clinical Study of Intravesical Ruvidar® in Patients With BCG‐Unresponsive Non‐Muscle Invasive Bladder Cancer ("NMIBC") Carcinoma In‐Situ ("CIS") or Patients Who Are Intolerant to BCG Therapy ("Study II")*, Clinicaltrials.Gov, (2025).

[16] C. Bhandari , S. Soma , M. Quaye , et al., “Predicting Head and Neck Tumor Nodule Responses to TLD1433 Photodynamic Therapy Using the Image‐Guided Surgery Probe ABY‐029,” Photochemistry and Photobiology 101 (2025): 1199–1210, 10.1111/php.14083. 40365701 PMC12353352

[17] R. Trondl , P. Heffeter , C. R. Kowol , M. A. Jakupec , W. Berger , and B. K. Keppler , “NK*P‐*1339, the First Ruthenium‐Based Anticancer Drug on the Edge to Clinical Application,” Chemical Science 5 (2014): 2925–2932, 10.1039/C3SC53243G.

[18] B. Happl , T. Balber , P. Heffeter , et al., “Synthesis and Preclinical Evaluation of BOLD‐100 Radiolabeled With Ruthenium‐97 and Ruthenium‐103,” Dalton Transactions 53 (2024): 6031–6040, 10.1039/D4DT00118D. 38470348

[19] B. Neuditschko , A. A. Legin , D. Baier , et al., “Interaction With Ribosomal Proteins Accompanies Stress Induction of the Anticancer Metallodrug BOLD‐100/KP1339 in the Endoplasmic Reticulum,” Angewandte Chemie International Edition 60 (2021): 5063–5068, 10.1002/anie.202015962. 33369073 PMC7986094

[20] Bold Therapeutics, Inc ., *A Phase 1b/2a Dose Escalation Study of BOLD‐100 in Combination With FOLFOX Chemotherapy in Patients With Advanced Solid Tumours*, Clinicaltrials.Gov, (2025).

[21] S. Top , A. Vessières , G. Leclercq , et al., “Synthesis, Biochemical Properties and Molecular Modelling Studies of Organometallic Specific Estrogen Receptor Modulators (SERMs), the Ferrocifens and Hydroxyferrocifens: Evidence for an Antiproliferative Effect of Hydroxyferrocifens on both Hormone‐Dependent and Hormone‐Independent Breast Cancer Cell Lines,” Chemistry–European Journal 9 (2003): 5223–5236. 10.1002/chem.200305024 14613131

[22] M. Kalabay , Z. Szász , O. Láng , et al., “Investigation of the Antitumor Effects of Tamoxifen and Its Ferrocene‐Linked Derivatives on Pancreatic and Breast Cancer Cell Lines,” Pharmaceuticals 15 (2022): 314, 10.3390/ph15030314. 35337112 PMC8950591

[23] V. Tomar , P. Kumar , D. Sharma , R. K. Joshi , and M. Nemiwal , “Anticancer Potential of Ferrocene‐Containing Derivatives: Current and Future Prospective,” Journal of Molecular Structure 1319 (2025): 139589, 10.1016/j.molstruc.2024.139589.

[24] C. Schmidt , M. Zollo , R. Bonsignore , A. Casini , and S. M. Hacker , “Competitive Profiling of Ligandable Cysteines in *Staphylococcus aureus* With an Organogold Compound,” Chemical Communications 58 (2022): 5526–5529, 10.1039/D2CC01259F. 35420608

[25] L. Skos , Y. Borutzki , C. Gerner , and S. M. Meier‐Menches , “Methods to Identify Protein Targets of Metal‐Based Drugs,” Current Opinion in Chemical Biology 73 (2023): 102257, 10.1016/j.cbpa.2022.102257. 36599256

[26] S. P. Fricker , “Cysteine Proteases as Targets for Metal‐Based Drugs,” Metallomics Integrated Biometal Science 2 (2010): 366–377, 10.1039/b924677k. 21072382

[27] E. Meggers , G. E. Atilla‐Gokcumen , H. Bregman , et al., “Exploring Chemical Space With Organometallics: Ruthenium Complexes as Protein Kinase Inhibitors,” Synlett 8 (2007): 1177–1189, 10.1055/s-2007-973893.

[28] R. Anand , J. Maksimoska , N. Pagano , et al., “Towards the Development of a Potent and Selective Organoruthenium Mammalian Sterile 20 Kinase Inhibitor,” Journal of Medicinal Chemistry 52 (2009): 1602–1611, 10.1021/jm8005806. 19226137 PMC2708071

[29] S. Blanck , T. Cruchter , A. Vultur , et al., “Organometallic Pyridylnaphthalimide Complexes as Protein Kinase Inhibitors,” Organometallics 30 (2011): 4598–4606, 10.1021/om200366r. 21918590 PMC3170819

[30] P. Xie , D. S. Williams , G. E. Atilla‐Gokcumen , et al., “Structure‐Based Design of an Organoruthenium Phosphatidyl‐inositol‐3‐kinase Inhibitor Reveals a Switch Governing Lipid Kinase Potency and Selectivity,” ACS Chemical Biology 3 (2008): 305–316, 10.1021/cb800039y. 18484710 PMC3618672

[31] H. Bregman , P. J. Carroll , and E. Meggers , “Rapid Access to Unexplored Chemical Space by Ligand Scanning Around a Ruthenium Center: Discovery of Potent and Selective Protein Kinase Inhibitors,” Journal of the American Chemical Society 128 (2006): 877–884, 10.1021/ja055523r. 16417378

[32] H. Johansson , Y. C. I. Tsai , K. Fantom , et al., “Fragment‐Based Covalent Ligand Screening Enables Rapid Discovery of Inhibitors for the RBR E3 Ubiquitin Ligase HOIP,” Journal of the American Chemical Society 141 (2019): 2703–2712, 10.1021/jacs.8b13193. 30657686 PMC6383986

[33] W. J. McCarthy , A. J. van der Zouwen , J. T. Bush , and K. Rittinger , “Covalent Fragment‐Based Drug Discovery for Target Tractability,” Current Opinion in Structural Biology 86 (2024): 102809, 10.1016/j.sbi.2024.102809. 38554479

[34] L. Boike , N. J. Henning , and D. K. Nomura , “Advances in Covalent Drug Discovery,” Nature Reviews Drug Discovery 21 (2022): 881–898, 10.1038/s41573-022-00542-z. 36008483 PMC9403961

[35] C. G. Parker , A. Galmozzi , Y. Wang , et al., “Ligand and Target Discovery by Fragment‐Based Screening in Human Cells,” Cell 168 (2017): 527–541, 10.1016/j.cell.2016.12.029. 28111073 PMC5632530

[36] V. M. Crowley , M. Thielert , and B. F. Cravatt , “Functionalized Scout Fragments for Site‐Specific Covalent Ligand Discovery and Optimization,” ACS Central Science 7 (2021): 613–623, 10.1021/acscentsci.0c01336. 34056091 PMC8155467

[37] F. Offensperger , G. Tin , M. Duran‐Frigola , et al., “Large‐Scale Chemoproteomics Expedites Ligand Discovery and Predicts Ligand Behavior in Cells,” Science 384 (2024): 1–12, 10.1126/science.adk5864. 38662832

[38] D. G. Hoch , D. Abegg , and A. Adibekian , “Cysteine‐Reactive Probes and Their Use in Chemical Proteomics,” Chemical Communications 54 (2018): 4501–4512, 10.1039/C8CC01485J. 29645055

[39] L. Goebel , M. P. Müller , R. S. Goody , and D. Rauh , “KRasG12C Inhibitors in Clinical Trials: A Short Historical Perspective,” RSC Medicinal Chemistry 11 (2020): 760–770, 10.1039/D0MD00096E. 33479673 PMC7549139

[40] J. M. Ostrem , U. Peters , M. L. Sos , J. A. Wells , and K. M. Shokat , “K‐Ras(G12C) Inhibitors Allosterically Control GTP Affinity and Effector Interactions,” Nature 503 (2013): 548–551, 10.1038/nature12796. 24256730 PMC4274051

[41] S. R. Thomas , T. Iellici , M. Park , et al., “A Gold‐PROTAC Degrades the Oncogenic Tyrosine Kinase MERTK: Insights Into the Degradome From a Steady‐State System,” ACS Chemical Biology 21 (2026): 170–186, 10.1021/acschembio.5c00860. 41486557 PMC12813982

[42] S. Swaminathan and R. Karvembu , “Dichloro Ru(II)‐*p‐*cymene‐1,3,5‐triaza‐7‐phosphaadamantane (RAPTA‐C): A Case Study,” ACS Pharmacology & Translational Science 6 (2023): 982–996, 10.1021/acsptsci.3c00085. 37470017 PMC10353064

[43] K. M. Backus , B. E. Correia , K. M. Lum , et al., “Proteome‐Wide Covalent Ligand Discovery in Native Biological Systems,” Nature 534 (2016): 570–574, 10.1038/nature18002. 27309814 PMC4919207

[44] A. J. Maurais and E. Weerapana , “Reactive‐Cysteine Profiling for Drug Discovery,” Current Opinion in Chemical Biology 50 (2019): 29–36, 10.1016/j.cbpa.2019.02.010. 30897495 PMC6584045

[45] H. Wilders , G. Biggs , S. M. Rowe , et al., “Expedited SARS‐CoV‐2 Main Protease Inhibitor Discovery Through Modular ‘Direct‐to‐Biology’ Screening,” Angewandte Chemie International Edition 64 (2025): e202418314, 10.1002/anie.202418314. 39630105

[46] J. Hess , M. Patra , V. Pierroz , et al., “Synthesis, Characterization, and Biological Activity of Ferrocenyl Analogues of the Anthelmintic Drug Monepantel,” Organometallics 35 (2016): 3369–3377, 10.1021/acs.organomet.6b00577.

[47] C. Mahe , O. Blacque , G. Gasser , V. Gandon , and K. Cariou , “N‐Metallocenyl Ynamides: Preparation, Reactivity, and Synthesis of Ansa[3]‐Ferrocenylamides,” Organic Letters 25 (2023): 624–629, 10.1021/acs.orglett.2c04169. 36688847

[48] E. Belhadj , A. El‐Ghayoury , T. Cauchy , M. Allain , M. Mazari , and M. Sallé , “Tetrathiafulvalene‐Based Phenanthroline Ligands: Synthesis, Crystal Structures, and Electronic Properties,” European Journal of Inorganic Chemistry 2014 (2014): 3912–3919, 10.1002/ejic.201402073.

[49] F. N. Castellano , J. D. Dattelbaum , and J. R. Lakowicz , “Long‐Lifetime Ru(II) Complexes as Labeling Reagents for Sulfhydryl Groups,” Analytical Biochemistry 255 (1998): 165–170, 10.1006/abio.1997.2468. 9451499

[50] M. C. Martos‐Maldonado , I. Quesada‐Soriano , F. García‐Maroto , A. Vargas‐Berenguel , and L. García‐Fuentes , “Ferrocene Labelings as Inhibitors and Dual Electrochemical Sensors of human Glutathione S‐Transferase P1‐1,” Bioorganic & Medicinal Chemistry Letters 22 (2012): 7256–7260, 10.1016/j.bmcl.2012.09.022. 23072868

[51] F. Wang , H. Chen , J. A. Parkinson , P. del Socorro Murdoch , and P. J. Sadler , “Reactions of a Ruthenium(II) Arene Antitumor Complex With Cysteine and Methionine,” Inorganic Chemistry 41 (2002): 4509–4523, 10.1021/ic025538f. 12184769

[52] G. S. Biggs , E. E. Cawood , A. Vuorinen , et al., “Robust Proteome Profiling of Cysteine‐Reactive Fragments Using Label‐Free Chemoproteomics,” Nature Communications 16 (2025): 1–14, 10.1038/s41467-024-55057-5. PMC11697256 39746958

[53] M. Kuljanin , D. C. Mitchell , D. K. Schweppe , et al., “Reimagining High‐Throughput Profiling of Reactive Cysteines for Cell‐Based Screening of Large Electrophile Libraries,” Nature Biotechnology 39 (2021): 630–641, 10.1038/s41587-020-00778-3. PMC8316984 33398154

[54] E. Weerapana , C. Wang , G. M. Simon , et al., “Quantitative Reactivity Profiling Predicts Functional Cysteines in Proteomes,” Nature 468 (2010): 790–795, 10.1038/nature09472. 21085121 PMC3058684

[55] E. V. Vinogradova , X. Zhang , D. Remillard , et al., “An Activity‐Guided Map of Electrophile‐Cysteine Interactions in Primary Human T Cells,” Cell 182 (2020): 1009–1026.e29, 10.1016/j.cell.2020.07.001. 32730809 PMC7775622

[56] “Pharos: Illuminating the Druggable Genome,” can be found under https://pharos.nih.gov (accessed 1 July 2025), (2025).

[57] M. Békés , D. R. Langley , and C. M. Crews , “PROTAC Targeted Protein Degraders: The Past Is Prologue,” Nature Reviews Drug Discovery 21 (2022): 181–200. 35042991 10.1038/s41573-021-00371-6 PMC8765495

[58] Z. Xiao , E. S. Gavriil , F. Cao , et al., “Identification of Actionable Targeted Protein Degradation Effector Sites Through Site‐Specific Ligand Incorporation‐Induced Proximity (SLIP),” Journal of the American Chemical Society 147 (2025): 21549–21559, 10.1021/jacs.5c01420. 40493711 PMC12203575

[59] J. T. Cruite , G. P. Dann , J. Che , et al., “Cereblon Covalent Modulation Through Structure‐Based Design of Histidine Targeting Chemical Probes,” RSC Chemical Biology 3 (2022): 1105–1110, 10.1039/D2CB00078D. 36128501 PMC9428674

[60] X. X. Yu , Y. Liu , Z. M. Mo , R. J. Luo , and W. K. Chen , “Exploring BIRC family Genes as Prognostic Biomarkers and Therapeutic Targets in Prostate Cancer,” Discover Oncology 16 (2025): 1–19. 40009266 10.1007/s12672-025-02002-7 PMC11865399

[61] L. Dietz , C. J. Ellison , C. Riechmann , et al., “Structural Basis for SMAC‐Mediated Antagonism of Caspase Inhibition by the Giant Ubiquitin Ligase BIRC6,” Science 379 (2023): 1112–1117, 10.1126/science.ade8840. 36758106

[62] S. S. Liu , T. X. Jiang , F. Bu , et al., “Molecular Mechanisms Underlying the BIRC6‐Mediated Regulation of Apoptosis and Autophagy,” Nature Communications 15, no. 1 (2024): 1–16. 10.1038/s41467-024-45222-1 PMC10827748 38291026

[63] K. C. Ehrlich , C. Baribault , and M. Ehrlich , “Epigenetics of Muscle‐ and Brain‐Specific Expression of KLHL Family Genes,” International Journal of Molecular Sciences 21 (2020): 8394, 10.3390/ijms21218394. 33182325 PMC7672584

[64] V. A. Gupta , G. Ravenscroft , R. Shaheen , et al., “Identification of KLHL41 Mutations Implicates BTB‐Kelch‐Mediated Ubiquitination as an Alternate Pathway to Myofibrillar Disruption in Nemaline Myopathy,” American Journal of Human Genetics 93 (2013): 1108–1117, 10.1016/j.ajhg.2013.10.020. 24268659 PMC3852928

[65] M. D. Petroski and R. J. Deshaies , “Function and Regulation of Cullin‐RING Ubiquitin Ligases,” Nature Reviews Molecular Cell Biology 6 (2005): 9–20, 10.1038/nrm1547. 15688063

[66] L. D. W. Hillier , T. A. Graves , R. S. Fulton , et al., “Generation and Annotation of the DNA Sequences of Human Chromosomes 2 and 4,” Nature 434 (2005): 724–731, 10.1038/nature03466. 15815621

[67] D. B. Grabarczyk , O. A. Petrova , L. Deszcz , et al., “HUWE1 Employs a Giant Substrate‐Binding Ring to Feed and Regulate Its HECT E3 Domain,” Nature Chemical Biology 17 (2021): 1084–1092, 10.1038/s41589-021-00831-5. 34294896 PMC7611724

[68] Z. Liu , J. R. Remsberg , H. Li , et al., “Proteomic Ligandability Maps of Spirocycle Acrylamide Stereoprobes Identify Covalent ERCC3 Degraders,” Journal of the American Chemical Society 146 (2024): 10393–10406, 10.1021/jacs.3c13448. 38569115 PMC11211653

[69] J. Jumper , R. Evans , A. Pritzel , et al., “Highly Accurate Protein Structure Prediction With AlphaFold,” Nature 596 (2021): 583–589, 10.1038/s41586-021-03819-2. 34265844 PMC8371605

[70] T. C. Terwilliger , D. Liebschner , T. I. Croll , et al., (2021), AlphaFold predictions: great hypotheses but no match for experiment.

[71] M. Karelina , J. J. Noh , and R. O. Dror , “How Accurately Can One Predict Drug Binding Modes Using AlphaFold Models?,” Elife 12 (2023): RP89386, 10.7554/eLife.89386.2. 38131311 PMC10746139

[72] I. G. Riziotis , A. J. M. Ribeiro , N. Borkakoti , and J. M. Thornton , “Conformational Variation in Enzyme Catalysis: A Structural Study on Catalytic Residues,” Journal of Molecular Biology 434 (2022): 167517, 10.1016/j.jmb.2022.167517. 35240125 PMC9005782

[73] I. Tolbatov and A. Marrone , “Reactivity of N‐Heterocyclic Carbene Half‐Sandwich Ru‐, Os‐, Rh‐, and Ir‐Based Complexes With Cysteine and Selenocysteine: A Computational Study,” Inorganic Chemistry 61 (2022): 746–754, 10.1021/acs.inorgchem.1c03608. 34894670

[74] M. Yoshimatsu , G. Toyokawa , S. Hayami , et al., “Dysregulation of PRMT1 and PRMT6, Type I Arginine Methyltransferases, Is Involved in Various Types of human Cancers,” International Journal of Cancer 128 (2011): 562–573, 10.1002/ijc.25366. 20473859

[75] A. C. e Silva , C. Y. C. Wu , C. T. Citadin , et al., “Protein Arginine Methyltransferases in Cardiovascular and Neuronal Function,” Molecular Neurobiology 57, no. 3 (2019): 1716–1732. 31823198 10.1007/s12035-019-01850-z PMC7062579

[76] J. H. Pyun , H. J. Kim , M. H. Jeong , et al., “Cardiac Specific PRMT1 Ablation Causes Heart Failure Through CaMKII Dysregulation,” Nature Communications 9, no. 1 (2018): 1–15, 10.1038/s41467-018-07606-y. PMC6269446 30504773

[77] B. Xie , J. Yu , C. Chen , and T. Shen , “Protein Arginine Methyltransferases From Regulatory Function to Clinical Implication in Central Nervous System,” Cellular and Molecular Neurobiology 45, no. 1 (2025): 1–21, 10.1007/s10571-025-01546-0. PMC12078925 40366461

[78] H. Zhou , Y. Wang , D. Wang , M. Zhang , K. Wang , and C. Liu , “PRMT1 Promotes Immune Escape in Hepatocellular Carcinoma by Regulating Arginine Methylation Modification of MYC Protein,” Epigenetics 20 (2025): 1–15, 10.1080/15592294.2025.2509044. PMC12101584 40401713

[79] M. S. Eram , Y. Shen , M. M. Szewczyk , et al., “A Potent, Selective, and Cell‐Active Inhibitor of Human Type I Protein Arginine Methyltransferases,” ACS Chemical Biology 11 (2016): 772–781, 10.1021/acschembio.5b00839. 26598975 PMC4798913

[80] Y. Xie , R. Zhou , F. Lian , et al., “Virtual Screening and Biological Evaluation of Novel Small Molecular Inhibitors Against Protein Arginine Methyltransferase 1 (PRMT1),” Organic & Biomolecular Chemistry 12 (2014): 9665–9673, 10.1039/C4OB01591F. 25348815

[81] D. Cheng , N. Yadav , R. W. King , M. S. Swanson , E. J. Weinstein , and M. T. Bedford , “Small Molecule Regulators of Protein Arginine Methyltransferases,” Journal of Biological Chemistry 279 (2004): 23892–23899, 10.1074/jbc.M401853200. 15056663

[82] A. Fedoriw , S. R. Rajapurkar , S. O'Brien , et al., “Anti‐Tumor Activity of the Type I PRMT Inhibitor, GSK3368715, Synergizes With PRMT5 Inhibition Through MTAP Loss,” Cancer Cell 36 (2019): 100–114.e25, 10.1016/j.ccell.2019.05.014. 31257072

[83] R. Taniguchi , Y. Moriya , N. Dohmae , et al., “Attenuation of Protein Arginine Dimethylation via *S*‐nitrosylation of Protein Arginine Methyltransferase 1,” Journal of Pharmacological Sciences 154 (2024): 209–217, 10.1016/j.jphs.2023.12.012. 38395522

[84] G. M. Morris , H. Ruth , W. Lindstrom , et al., “AutoDock4 and AutoDockTools4: Automated Docking With Selective Receptor Flexibility,” Journal of Computational Chemistry 30 (2009): 2785–2791, 10.1002/jcc.21256. 19399780 PMC2760638

[85] M. L. A. Hakkennes , F. Buda , and S. Bonnet , “MetalDock: An Open Access Docking Tool for Easy and Reproducible Docking of Metal Complexes,” Journal of Chemical Information and Modeling 63 (2023): 7816–7825, 10.1021/acs.jcim.3c01582. 38048559 PMC10751784

[86] C. Thiebaut , L. Eve , C. Poulard , and M. Le Romancer , “Structure, Activity, and Function of PRMT1,” Life 11 (2021): 1147, 10.3390/life11111147. 34833023 PMC8619983

[87] “ProteomeCentral Datasets,” can be found under https://proteomecentral.proteomexchange.org/ (accessed 12 June 2025), (2025).

[88] Y. Perez‐Riverol , J. Bai , C. Bandla , et al., “The PRIDE Database Resources in 2022: A Hub for Mass Spectrometry‐Based Proteomics Evidences,” Nucleic Acids Research 50 (2022): D543–D552, 10.1093/nar/gkab1038. 34723319 PMC8728295

