# Supplementary material for: Proteome‐Wide Target Identification Using Reactive Metallo‐Scaffolds (r‐mS): A Platform for Metallodrug Discovery
Source: Angew Chem Int Ed Engl. 2026 Jul 10;65(38):e8867549. doi: 10.1002/anie.8867549 (PMC13573063; doi:10.1002/anie.8867549)
Supplement: Supplementary file 1 [file ANIE-65-e8867549-s001.docx]

Supporting Information

**Proteome-Wide Target Identification Using Reactive Metallo-Scaffolds (*r*-mS) : A Platform for Metallodrug Discovery**

Jessica E. Waters,^[a,b]^ Harry Wilders,^[c]^ George S. Biggs,^[c]^ Mika Kintzel,^[a,b]^, Emma E. Cawood,^[c]^ Jonathan Bailey,^[a]^ Sarah Maslen,^[d]^ Yew Mun Yip,^[e]^ Mason Wakley,^[a]^ Ioannis G. Riziotis,^[c]^ Thomas W. Rees,^[a]^ Joanna Redmond,^[e]^ J. Mark Skehel,^[d]^ David House,^[c]^ Jacob Bush^*^,^[c]^ Jeannine Hess^*^,^[a,b]^

[a] The Biological Inorganic Chemistry Laboratory, The Francis Crick Institute, 1 Midland Road, London, NW1 1AT, United Kingdom.

[b] Department of Chemistry, King’s College London, Britannia House, 7 Trinity Street, London, SE1 1DB, United Kingdom.

[c] Crick-GSK Biomedical LinkLabs, Gunnels Wood Road, Stevenage, Hertfordshire, SG1 2NY, United Kingdom.

[d] Proteomics Science Technology Platform, The Francis Crick Institute, 1 Midland Road, London, NW1 1AT, United Kingdom.

[e] Chemical Biology Science Technology Platform, The Francis Crick Institute, 1 Midland Road, London, NW1 1AT, United Kingdom.

*Corresponding Author (jacob.x.bush@gsk.com, jeannine.hess@crick.ac.uk or jeannine.hess@kcl.ac.uk).

**Table of Contents**

[Supplementary Materials and Methods	4](#_Toc222287721)

[S1. Stability of *r*-mS Methods	6](#_Toc222287722)

[S2. Chemoproteomics Methods	8](#_Toc222287723)

[S3. Interactions of *r*-mS-2 with Amino Acids	11](#_Toc222287724)

[Intrinsic Reactivity of Compounds with *L*-Cysteine	11](#_Toc222287725)

[S4 Computational Workflow for Cysteine Environment Descriptor Analysis	12](#_Toc222287726)

[S5 PRMT1 Expression and Purification	13](#_Toc222287727)

[S6 Incubation of *r*-mS-2 with PRMT1	18](#_Toc222287729)

[S7 HMT Assay Protocol for PRMT1	19](#_Toc222287730)

[S8 Computationally-Predicted Binding Poses & Docking Procedure	20](#_Toc222287731)

[Synthesis and Characterisation	21](#_Toc222287732)

[Bis(2,2’-bipyridyl)(2-chloro-*N*-1,10-phenanthrolin-5-yl-acetamide)ruthenium(II) hexafluorophosphate *r*-mS-1	22](#_Toc222287733)

[Ruthenium(II)($\eta$^6^-*p*-cymene)(2-chloro-N-1,10-phenanthrolin-5-ylacetamide)chloride, *r*-mS-2	25](#_Toc222287734)

[Ferrocenecarboxaldehyde Oxime	28](#_Toc222287735)

[Aminomethylferrocene, 10	29](#_Toc222287736)

[*N*-(ferrocenemethy)lchloroatemide, *r*-mS-3	31](#_Toc222287737)

[2-chloro-*N*-1,10-phenanthrolin-5-yl-acetamide, Lig-1	33](#_Toc222287738)

[Ruthenium(II)($\eta$^6^-hexamethylbenzene)(2-chloro-N-1,10-phenanthrolin-5-ylacetamide)chloride, *r*-mS-4	35](#_Toc222287739)

[Ruthenium(II)($\eta$^6^-benzene)(2-chloro-N-1,10-phenanthrolin-5-ylacetamide)chloride, *r*-mS-5	38](#_Toc222287740)

[Osmium(II)($\eta$^6^-*p*-cymene) dichloride, 11	41](#_Toc222287741)

[Osmium(II)($\eta$^6^-*p*-cymene)(2-chloro-N-1,10-phenanthrolin-5-ylacetamide)chloride, *r*-mS-6	43](#_Toc222287742)

[Iridium(III)($\eta$^5^-cyclopentadienyl)(2-chloro-N-1,10-phenanthrolin-5-ylacetamide)chloride, *r*-mS-7	46](#_Toc222287743)

[Ruthenium(II)($\eta$^6^-*p*-cymene)(5-methyl-1,10-phenanthroline)chloride, compound 9	49](#_Toc222287744)

[S9 *r*-mS Scaffold Nomenclature and Structures	52](#_Toc222287745)

[S10 PMI Plot of *r*-mS	53](#_Toc222287746)

[S11 Stability of *r*-mS	54](#_Toc222287747)

[S12 3-Point Concentration Data for First Generation *r*-mS	60](#_Toc222287748)

[S13 Reactivity between *r*-mS-2 and Amino Acids	61](#_Toc222287749)

[S14 Structural Feature Enrichment Analysis	65](#_Toc222287750)

[S15 8-Point Concentration-Response Curves: *r*-mS and PRMT1_Cys119	66](#_Toc222287751)

[S16 *p*TE50 Values of Second Generation *r*-mS	67](#_Toc222287752)

[S17 Intact Protein LC-MS Data of PRMT1	68](#_Toc222287753)

[S18 Electrostatic Representation of *r*-mS-2-docked PRMT1	70](#_Toc222287754)

[References	71](#_Toc222287755)

# Supplementary Materials and Methods

All synthetic procedures were carried out under ambient conditions unless otherwise stated. All reagents and solvents were used as received or dried over molecular sieves. Reagents were purchased from commercial suppliers: 1,10-phenanthroline-5-amine was purchased from Fluorochem and all dimeric metal precursors ([(*η*^6^-*p*-cymene)RuCl_2_]_2_, [(*η*^6^-hexamethylbenzene)RuCl_2_]_2_, [(*η*^6^-*benzene*)RuCl_2_]_2_ and [(*η*^5^-pentamethylcyclopentadienyl)IrCl_2_]_2_ were purchased either from Thermo Scientific, Alfa Aesar or Merck. Complex **12** - ([(*η*^6^-*p*-cymene)OsCl_2_]_2_ - was synthesised following literature procedures. ^[1]^ Reaction flasks and storage vials containing reactive scaffolds were wrapped in aluminium foil and stored at -20 °C. Column chromatography was performed using a Biotage Selekt equipped with Biotage Sfär column packed with silica gel 60. Eluent mixtures are expressed as volume to volume ((v/v)) ratios. A combination of Thin-layer chromatography (TLC) and Liquid chromatography mass spectrometry (LC-MS) analysis was used to monitor reactions. TLC was performed using silica gel 60 F-254 (Merck) plates with detection of spots being achieved by exposure to UV light. All tested compounds possessed a purity of at least 95% as determined by LC-MS analysis. LC-MS was carried out using a Waters Acquity UPLC H-Class system, with samples run on a solvent gradient from 0 to 95% acetonitrile in water (+ 0.1% formic acid) over 4 min. Peaks corresponding to the desired product are described, including the retention time (rt) and % purity by integration. High-resolution mass spectra (HR-MS) using electron spray ionisation (ESI) were recorded on a Waters Xevo G2-XS QTof instrument (resolution of 20,000 FWHM) coupled to an Acquity UPLC system using an Acquity UPLC BEH C18 column (1.7 $\mu$m, 2.1 x 100 mm)). H_2_O with 0.1 % formic acid (solvent A) and 95% acetonitrile and 5 % water with 0.1 % formic acid (solvent B), both LC-MS-grade, were used as the mobile phase at a flow rate of 0.5 mL/min. The gradient was programmed as follows: 0−3min, isocratic 95% A (5% B); 3−17 min, linear gradient from 95% A (5% B) to
0% A (100% B); 17−25 min, isocratic 0% A (100% B), unless otherwise stated. Nuclear magnetic resonance (NMR) spectra were recorded at 298 K on a 400 MHz Avance III HD Smart Probe Spectrometer (^1^H at 400.1 MHz or 600 MHz; ^13^C at 100.6 MHz) unless otherwise stated. The chemical shifts, $\delta\partial$, are reported in parts per million (ppm) to the nearest 0.01 ppm, with coupling constants (J) recorded in Hertz (Hz). Data are reported in the order: (i) chemical shift, (ii) multiplicity, abbreviated as s (singlet), d (doublet), t (triplet), q (quartet), sept (septet), m (multiplet) and combinations thereof for highly coupled systems, (iii) coupling constant(s), (iv) integration and (v) assignment. ^1^H and ^13^C NMR spectra were referenced to residual solvent peaks, and unambiguous assignments were made on the basis of 2D NMR experiments: homonuclear correlation spectroscopy (^1^H-^1^H COSY), heteronuclear single quantum correlation spectroscopy (^1^H-^13^C HSQC) and heteronuclear multiple-bond correlation spectroscopy (^1^H-^13^C HMBC) and ^19^F {^1^H} NMR spectroscopy. For simplification, Figure S1 below shows the labelling scheme for the NMR spectroscopy assignments used for the reactive-scaffold systems.

**Figure S1**: Labelling scheme for ^1^H NMR spectroscopy assignments.

## S1. Stability of *r*-mS Methods

The stability of the *r*-mS series was examined in the radioimmunoprecipitation assay lysis buffer (RIPA, constituents listed in Table S1, below) to simulate the conditions in chemoproteomics sample preparation. Acetaminophen was obtained from Sigma-Aldrich and used as an internal standard for ***r*-mS-1**. Caffeine was obtained from Sigma-Aldrich and used as an internal standard for ***r*-mS-2** and ***r*-mS-3**. For each experiment, fresh stocks of each *r*-mS (10 mM, DMSO-*d*_6_) were prepared and isolated from light. Each *r*-mS sample was then prepared in triplicate from a 10 mM DMSO-*d*_6_ stock to 100 $\mu$M in RIPA lysis buffer, and kept in the dark at 25 °C for a total of 60 min with continuous shaking at 600 rpm. At various timepoints, aliquots of the incubate were taken, spiked with their respective internal standard and their UPLC chromatogram obtained. Analysis by LC-MS was performed on a Xevo G2-XS QTof mass spectrometer coupled to an Acquity UPLC system using an Acquity BEH C18 column (1.7 $\mu$m, 2.1 x 100 mm)). H_2_O with 0.1% formic acid (solvent A) and 95% acetonitrile and 5% water with 0.1% formic acid (solvent B), were used as the mobile phase at a flow rate of 0.5 mL/min. 5 $\mu$L of sample was injected, and the gradient was programmed as follows: 0−3min, isocratic 95% A (5% B); 3−17 min, linear gradient from 95% A(5% B) to 0% A (100% B); 17−25 min, isocratic 0% A (100% B). UV absorption was measured at 260 nm. Chromatographic data were then extracted, and data replotted using GraphPad Prism (Figure S33-35). Degradation of each scaffold was calculated using the following three equations above Table S1.

$\frac{Area (r-mS, t0)}{Area (Standard, t0)}=Ratio (t0)$	$\frac{Area (r-mS,tx)}{Area (Standard,tx)}=Ratio (tx)$	$\frac{Ratio(tx)}{Ratio(t0)}*100$

**Table S1**: Constituents of RIPA lysis buffer stock used in chemoproteomics sample preparation and incubations. Note: all detergents were excluded from incubation experiments due to their incompatibility with LC-MS instruments. The stock was diluted 5-fold prior to experimental use.

| **Stock** | **Volume of Stock** | **Final Concentration** |
| --- | --- | --- |
| **1 M HEPES pH 8.0** | 5 mL | 50 mM |
| **5 M NaCl** | 3 mL | 150 mM |
| **IGEPAL (Sigma, 18896)** | 1 mL | 1% |
| **20% SDS (Sigma, L4509)** | 500 $\mu$L | 0.1% |
| **10% Na-deoxycholate (Sigma, D6750)** | 5 mL | 0.5% |
| **Milli-Q Water** | 85.5 mL |  |
| **Protease Inhibitor Cocktail (Sigma-Aldrich, P8340)** | 1:100 |  |

## S2. Chemoproteomics Methods

All chemoproteomic sample preparation and data analysis was performed according to literature methods, which are visually summarised in Figure 1b.^[2]^

**Compounds**

All chloroacetamide scaffolds (***r*-mS-1** to ***r*-mS-7** and **Lig-1**) were synthesised and stored as solids at -20 °C. Iodoacetamide desthiobiotin (IA-DTB) was synthesised according to literature reports.^[3]^ Fresh stock was prepared from solid at a concentration of 50 mM in dimethyl sulfoxide (DMSO) and used immediately.

**Cell culture**

Adherent HEK293T cells were maintained at 37 °C, 5% CO_2_ in Dulbecco’s Modified Eagle Medium (Gibco, 41966-029) supplemented with 10% ((v/v)) fetal bovine serum (Gibco, 10270-106) and 1% (v/v) penicillin–streptomycin–glutamine (Gibco, 10-378-016).

**Lysate Compound Treatment**

Cells (pre-washed twice with PBS) were suspended in RIPA lysis buffer (150 mM NaCl, 1.0% IGEPAL CA-630 (Sigma Aldrich, I8896), 0.5% sodium deoxycholate (Sigma Aldrich, D6750), 0.1% SDS (Fisher Scientific, 10607633), 50 mM HEPES pH 8.0) containing protease inhibitor cocktail (Sigma Aldrich, P1860). Samples were sonicated with a Branson probe sonicator (3-5 × 2 s pulses, 10% amplitude) and filtered through a 0.22 µM filter. The protein concentration of the lysate was determined using a Pierce™ BCA assay kit (Thermo Scientific, 23227) according to manufacturer’s instructions. Lysate was diluted to the desired concentration and used on the day of lysis.

For cell lysate treatment, compounds were first prepared at an appropriate concentration in DMSO (2 μL) in a 96-well plate. To this, cell lysate containing 200 μg of protein (1 μg/μL) was added to each well and the plates were then shaken on a Thermomixer (room temperature, 600 rpm, 1 h).

**IA-DTB treatment**

IA-DTB (500 µM, 25 °C, 600 rpm, 1 h, dark) was added from a fresh DMSO stock to wells containing compound-treated cell lysates. Following treatment, samples were reduced with dithiothreitol (DTT, Thermo Scientific, R0861) (5 mM, 25 °C, 600 rpm, 30 min) and alkylated with iodoacetamide (Thermo Scientific, 122270250) (10 mM, 25 °C, 600 rpm, 30 min, dark).

**Protein Clean-Up**

Sample preparation and removal of excess small molecules was performed *via* glass-bead assisted SP4 precipitation according to previously reported methods.^[2]^

**Evotip Sample Loading**

Prepared samples were re-dissolved in Optima™ 0.1% (*v*/*v*) formic acid in water (100 µL/well) on a Thermomixer (25 °C, 600 rpm, 5 min). Evotips (Evosep, EV2011) were conditioned according to manufacturer’s instructions. Approximately 200 ng (the desired loading) of each peptide mixture spiked with iRT peptides (Biognosys, Ki-3002-1) was loaded onto a conditioned Evotip and queued on an Evosep One liquid chromatography system with the pre-defined 60 SPD (samples per day) method and the corresponding Evosep Performance column (Evosep, EV1137). Mobile phases A and B were 0.1%(*v*/*v*) ) formic acid in water and 0.1% (*v*/*v*) formic acid in acetonitrile, respectively.

**Mass Spectrometry: General Methods**

The Evosep One was coupled online to a hybrid trapped ion mobility spectrometry (TIMS) quadrupole time of flight (TOF) mass spectrometer (Bruker timsTOF Pro 2) *via* a captive spray nano-electrospray ion source. Mass spectrometry methods were adopted from those described previously.^[2]^

**Chemoproteomics Data Analysis**

Mass spectrometry raw files for chemoproteomics were analysed using Spectronaut (version 16.0.220606.53000). Data analysis from the compound screen was performed as previously stated.^[2]^

Data were searched in Spectronaut against a previously reported in-house spectral library. This library was generated from IA-DTB modified HEK293T lysate, according to previous literature reports.^[2]^

**Chemical and physical properties of the Reactive Metallo-Scaffold Series**

Normalised principal moment of inertia ratios (NPRs) were calculated within Molecular Operating Environment (MOE: Chemical Computing Group, version: 2019.0101).^[4]^ Principal moment of inertia (PMI) values were then calculated from the resulting PMI values. PMI for metal complexes were based on modified crystal structures. Crystal structures were accessed via the CCDC and modified using Biovia Discovery Studio Visualizer. For PMI calculations of organic molecules, a 3D sdf file was generated from the SMILES String using Open Babel 3.1.0.^[5]^

## S3. Interactions of *r*-mS-2 with Amino Acids

In order to assess the preferential ligandability of cysteines for metal-based chloroacetamides, ***r*-mS-2** was incubated with various amino acids (including *L*-cysteine) and its reactivity/rate of consumption assessed by LC-MS. Caffeine was obtained from Sigma-Aldrich and used as an internal standard. For each experiment, fresh stocks of each compound, (10 mM, DMSO-*d*_6_) were prepared and isolated from light.

***r*-mS-2** was incubated in a 1:1 molar ratio with amino acids with side chains of various chemical properties (*L*-arginine, *L*-histidine, *L*-aspartic acid, *L*-asparagine, *L*-glutamic acid, *L*-cysteine, *L*-methionine and *L*-lysine) in RIPA lysis buffer at pH 8.0 (2% (*v*/*v*) DMSO) for a total of 90 min in the dark with continuous shaking at 600 rpm. During this time, the solution was analysed at intervals of 10 min by LC-MS with 5 $\mu$L of sample injected, and identical mobile phase and gradient conditions used as above for stability experiments. UV absorption was measured at 290 nm. Chromatographic data were then extracted, and data replotted using GraphPad Prism. Calculations to determine the percentage of each *r*-mS remaining, and thus concomitant formation of ***r*-mS-2** -amino acid adduct, were performed as follows:

$\frac{Area (r-mS, t0)}{Area (Standard, t0)}=Ratio (t0)$	$\frac{Area (r-mS,tx)}{Area (Standard,tx)}=Ratio (tx)$	$\frac{Ratio(tx)}{Ratio(t0)}*100$

### Intrinsic Reactivity of Compounds with *L*-Cysteine

Compounds were incubated in a 1:1 molar ratio with *L*-cysteine ([Compound]_0_ = 500 µM) in RIPA lysis buffer at pH 8.0 (5% (*v*/*v*) DMSO) for a total of 90 min in the dark with continuous shaking at 600 rpm.

During this time, the solution was analysed at intervals of 10 min by LC-MS with 5 $\mu$L of sample injected, and identical mobile phase and gradient conditions used as above for stability experiments. UV absorption was measured at 260 nm. Chromatographic data were then extracted. Percentage compound remaining was calculated as described above (Section S3), and the concentration of compound at a given timepoint was calculated according to the following equation:

$$\left[ Compound \right]_{t}= \left[ Compound \right]_{0}* \frac{\% Remaining}{100}$$

The reciprocal of [Compound]_t_ was plotted for each timepoint, a linear regression was fitted, and the slope of the resultant fit (*k*) was extracted. The half-life for each compound was then calculated according to the following equation:

$t_{1/2} \left( s \right)= \frac{1}{k* {[Compound]}_{0}}$

## S4 Computational Workflow for Cysteine Environment Descriptor Analysis

To calculate structural descriptors for the feature enrichment analysis, we used off-the-shelf algorithms combined with in-house *Python* scripts, using as input the same canonical protein sequences from UniProt^[6]^ used for building and searching our spectral library, as well as their respective AlphaFold2^[7]^ predicted models. Cysteine conservation was extracted from a Position-Specific Scoring Matrix (PSSM) generated by running two iterations of PSI-BLAST^[6]^ over the Swiss-Prot database.^[7]^ Secondary structure propensity and sequence-derived solvent accessibility (pred_sasa) were predicted by a deep learning algorithm implemented in SPINE X,^[6]^ using the same PSSM matrix. pKa, buried-ness, hydrogen bonding and number of Coulomb interactions were calculated using PROPKA3^[6]^ and solvent accessibility (SASA) was calculated with the Shrake-Ruple algorithm^[8]^ on protein structures. pPSE values were collected from the extended data of White et al.^[8]^ For the environment descriptors, the same algorithms were applied on residues within a spherical area around the Cys thiol (4, 6, 8 and 10Å away), by using a *k-d* tree-based nearest neighbour searching algorithm implemented in the *Biopython* package.^[9]^ As “reactive” residues we define Cys, Asp, Glu, His, Lys, Tyr, Arg, Ser and Thr, and as “non-reactive” we define Gly, Ala, Val, Leu, Ile, Met, Pro, Trp, Phe, Asn, Gln. Binding pocket descriptors were calculated with Fpocket^[7]^ using 3Å minimum and 5Å maximum alpha-sphere sizes, and a 1.5Å maximum distance between the endpoint sulfur atom electron cloud edge and the closest pocket vertex edge as a criterium to define a cysteine residue as pocketed.

## S5 PRMT1 Expression and Purification

**Cloning of the Human PRMT1 Gene into the pNIC-Bsal Expression Vector**

A truncated version of the human PRMT1 gene, lacking the first 19 amino acids at the N-terminus,^[10]^ was codon-optimized for *Escherichia coli* (*E. coli*) expression and synthesized by Thermo Fisher Scientific. This N-terminal truncation has been shown to enhance PRMT1 solubility. For cloning into the pNIC-Bsal expression vector, primers were designed with complementary overhangs: forward primer (CC TTA AGA AGG AGA TAT ACT) and reverse primer (GCA GAG AAC CTC TAC TTC CA ATC). The synthesized gene/insert construct and corresponding amino acid sequence is presented in Figure S2-A. The insert was subcloned into the pNIC-Bsal expression vector *via* isothermal assembly. First, the pNIC-Bsal vector was linearized using BsaI-HF (NEB), and the insert was diluted in Milli-Q H₂O to a final concentration of 10 ng/µL (0.042 pmol), while the linearized pNIC-Bsal vector was adjusted to 50 ng/µL (0.02 pmol). The isothermal assembly mix included: 3 µL insert, 1.5 µL vector (2:1 molar ratio), 5.5 µL MQH₂O, and 10 µL NEBuilder HiFi DNA Assembly Master Mix (NEB). A negative control without NEBuilder HiFi DNA Assembly Mix was prepared. The reaction mixtures were gently mixed by pipetting, incubated at 50 °C for 30 min, and then placed on ice. For transformations, 2 µL of the assembly product was pipetted into 50 µL chemically competent *E. coli* NEB5α cells (NEB) and incubated on ice for 30 min. Next, the cells were heat shocked at 42 °C for 60 s, and returned to ice for 2 min. Afterward, 1 mL of Super Optimal broth with Catabolite repression (SOC) medium was added, and the cells were incubated at 37 °C with shaking (180 rpm) for 1 h. Next, 200 µL of the cell suspension was plated on Lysogeny Broth (LB)-agar supplemented with 50 µg/mL kanamycin and incubated overnight at 37 °C. Several resulting colonies were picked and used to inoculate 10 mL LB medium supplemented with 50 µg/mL kanamycin in 50 mL Falcon tubes. Cultures were grown for 21 h at 37 °C with shaking at 180 rpm. Plasmids were isolated using a plasmid mini-prep kit (Qiagen) using Qiagen’s protocol. Sequencing analysis (Eurofins) confirmed the successful insertion of the entire synthesised protein arginine methyltransferase 1 (PRMT1) insert into the pNIC vector. The final expression construct is depicted in Figure S2-B.

**Expression of PRMT1 in *E. coli* BL21 STAR (DE3) cells**

The sequence confirmed PRMT1-pNIC plasmid was transformed into E. coli BL21 STAR (DE3) cells via heat shock, as previously described. A starter culture was prepared by inoculating a single colony from the transformation LB-agar plate into 50 mL LB medium with 50 µg/mL kanamycin in a 250 mL flask, followed by incubation at 37 °C with shaking at 180 rpm for 18 h. This starter culture (5 mL) was then used to inoculate eight 2000 mL flasks containing 500 mL Terrific Broth (TB) with 50 µg/mL kanamycin. The cultures were grown at 37 °C, 180 rpm until reaching an OD₆₀₀ of approximately 0.6, after which they were transferred to a 20 °C incubator. Protein expression was induced with 0.5 mM isopropyl β-D-1-thiogalactopyranoside, IPTG, and cultures were incubated at 20°C, 180 rpm for 18 h. Successful expression of PRMT1 was confirmed by sodium dodecyl-sulfate polyacrylamide gel electrophoresis, SDS-PAGE. Following expression, cells were harvested by centrifugation at 4,000 rpm for 20 min at 4 °C, resuspended in lysis buffer (50 mM Tris-HCl pH 7.5, 250 mM NaCl, 10 mM imidazole, 5% ((v/v)) glycerol, 1 mM tris(2-carboxyethyl)phosphine (TCEP), flash-frozen in liquid nitrogen, and stored at -80 °C.

**Purification of PRMT1**

Cells were thawed at room temperature, supplemented with two complete protease inhibitor tablets (Roche) and 20 µL Benzonase Nuclease (Sigma), and then lysed using a probe sonicator (50% amplitude; 12 s on, 48 s off, for a total of 12 min on). During sonication, cells were kept on ice. The lysate was clarified by centrifugation at 40,000 rpm for 1 h at 4 °C, and the supernatant was transferred to a chilled 250 mL beaker. For nickel nitrilotriacetic acid, Ni-NTA chromatography, 5 mL of PurKine His-tag Ni-NTA resin (Abbkine), pre-equilibrated with lysis buffer containing 20 mM imidazole, was added to the supernatant, and binding was carried out for 2 h at 4°C with gentle mixing on a magnetic stirrer. The mixture was then applied to a 25 mL gravity column (Bio-Rad), and the flow-through was collected. The resin was washed sequentially with 50 mL lysis buffer + 20 mM imidazole, followed by 50 mL lysis buffer + 40 mM imidazole. Bound protein was eluted using lysis buffer + 300 mM imidazole, and fractions were analyzed by SDS-PAGE. Most of the PRMT1 protein eluted in the 300 mM imidazole fraction. To remove the N-terminal His₆-tag, TEV protease (TEVp) was added to a final concentration of 30 μg/mL (1 μM), and the sample was transferred to a Spectra/Por 1 dialysis membrane (6–8 kDa MWCO; Spectrum Labs) for overnight dialysis against 1 L of dialysis buffer (50 mM Tris-HCl pH 7.5, 250 mM NaCl, 5% (v/v) glycerol, 1 mM TCEP) to remove imidazole and facilitate tag cleavage. After TEVp cleavage, residual uncleaved protein, TEVp, and the cleaved His₆-tag were removed by binding to 2 mL PurKine His-tag Ni-NTA resin (Abbkine) pre-equilibrated with 20 mL dialysis buffer containing 10 mM imidazole. The sample was incubated with the resin for 2 h, then loaded onto a 25 mL gravity column (Bio-Rad), and the flow-through containing the cleaved PRMT1 was collected. The cleaved protein was concentrated to 7 mg/mL using a Vivaspin-20 ultrafiltration unit (10,000 Da MWCO; Sartorius) at 3,000 g in 10-min intervals, with gentle pipetting between intervals to homogenize. Protein concentration was determined using a NanoDrop spectrophotometer (PRMT1 ε280 nm = 55,810 M⁻¹ cm⁻¹, MW = 40,625 Da). The purified protein was aliquoted into 50 µL portions, flash-frozen in liquid nitrogen, and stored at −80 °C. Purity was assessed by SDS-PAGE, and identity and integrity were confirmed by intact mass spectrometry, with measured molecular weights within 0.001% of the expected mass based on the primary amino acid sequence.

**A.** DNA Sequence of *E. coli*-optimised Human PRMT1

CCGAGAACCTGTACTTCCAATCC (Forward primer)

AATGGTATGAGCCTGCAGCCTCCGCTGGAAGAAGTTAGCTGCGGTCAGGCAGAAAGCAGCGAAAAACCGAATGCAGAAGATATGACCAGCAAAGATTATTACTTCGATAGCTATGCCCATTTTGGCATCCATGAAGAAATGCTGAAAGATGAAGTTCGTACCCTGACCTATCGTAACAGCATGTTTCATAATCGCCACCTGTTCAAAGATAAAGTGGTTCTGGATGTTGGTAGCGGCACCGGTATTCTGTGTATGTTTGCAGCAAAAGCCGGTGCACGTAAAGTTATTGGTATTGAATGTAGCAGCATCAGCGATTATGCCGTGAAAATTGTGAAAGCCAACAAACTGGATCATGTGGTGACCATTATCAAAGGCAAAGTTGAAGAAGTTGAACTGCCGGTTGAAAAAGTGGATATCATTATTAGCGAATGGATGGGCTATTGCCTGTTTTATGAAAGTATGCTGAATACCGTTCTGTACGCACGTGATAAATGGCTGGCACCGGATGGTCTGATTTTTCCGGATCGTGCAACCCTGTATGTTACCGCAATTGAAGATCGTCAGTACAAGGATTACAAAATCCATTGGTGGGAAAACGTGTATGGCTTTGATATGAGCTGCATTAAAGATGTGGCAATCAAAGAACCGCTGGTTGATGTTGTTGATCCGAAACAGCTGGTTACCAATGCATGTCTGATTAAAGAGGTGGATATCTACACCGTGAAAGTGGAAGATCTGACATTTACCAGTCCGTTTTGTCTGCAGGTTAAACGCAATGATTATGTTCATGCACTGGTGGCCTATTTCAACATTGAATTTACCCGTTGTCATAAACGCACCGGTTTTAGCACCAGTCCGGAAAGCCCGTATACACATTGGAAACAGACCGTGTTTTACATGGAAGATTATCTGACCGTGAAAACGGGTGAAGAAATTTTTGGCACCATTGGTATGCGTCCGAATGCCAAAAACAATCGTGATCTGGATTTTACCATCGACCTGGATTTCAAAGGTCAGCTGTGTGAACTGAGCTGTAGCACCGATTATCGTATGCGT

TAACAGTAAAGGTGGATACGGATCCG (Reverse primer)

**B.** PRMT1 Expression Construct

M**HHHHHH**SSGVDLGT*ENLYFQ/S*NGMSLQPPLEEVSCGQAESSEKPNAEDMTSKDYYFDSYAHFGIHEEMLKDEVRTLTYRNSMFHNRHLFKDKVVLDVGSGTGILCMFAAKAGARKVIGIECSSISDYAVKIVKANKLDHVVTIIKGKVEEVELPVEKVDIIISEWMGYCLFYESMLNTVLYARDKWLAPDGLIFPDRATLYVTAIEDRQYKDYKIHWWENVYGFDMSCIKDVAIKEPLVDVVDPKQLVTNACLIKEVDIYTVKVEDLTFTSPFCLQVKRNDYVHALVAYFNIEFTRCHKRTGFSTSPESPYTHWKQTVFYMEDYLTVKTGEEIFGTIGMRPNAKNNRDLDFTIDLDFKGQLCELSCSTDYRMR

**Figure S2:** DNA and amino acid sequence of the PRMT1 recombinant construct. **A**. DNA sequence of E. coli-optimised PRMT1 subcloned into pNIC for recombinant expression. **B**. Amino acid expression construct, containing an N-terminal His_6_-tag (bold) and a TEV cleavage site (italiics).

## S6 Incubation of *r*-mS-2 with PRMT1

Protein samples (6.8 µg/µL) were incubated with ***r*-mS-1** (100 mM) in RIPA lysis buffer for 2 h at room temperature with shaking at 600 rpm in the dark. Proteins were digested with trypsin at an enzyme-to-substrate ratio of both 1:50 and 1:10, for 2 h at room temperature and further digested overnight at 37 °C following addition of trypsin at a ratio of 1:20.

Peptides were diluted in 1% (*v*/*v*) formic acid and 2% (*v*/*v*) acetonitrile and analysed by nano-scale capillary LC-MS/MS using a Vanquish Neo UPLC (ThermoScientific Dionex, USA) to deliver a flow of approximately 250 nL/min. A PepMap Neo C18 5 μm, 300 μm x 5 mm nanoViper (ThermoScientific Dionex, USA) trapped the peptides before separation on a 50 cm EASY‐Spray column (50 cm x 75 µm ID, PepMap C18, 2 µm particles, 100 Å pore size: ThermoScientific, USA). Peptides were eluted with a gradient of acetonitrile over 30 min. The analytical column outlet was directly interfaced via a nano-flow electrospray ionisation source, with a quadrupole Orbitrap mass spectrometer (Orbitrap Exploris 480, ThermoScientific, USA). MS data were acquired in data-dependent mode using a top 20 method, where ions with a precursor charge state of 1+ was excluded. High-resolution full scans (R=60000, m/z 380-1600) were recorded in the Orbitrap followed by higher energy collision dissociation (HCD) using normalized collision energy of 30% for the 20 most intense MS peaks. The fragment ion spectra were acquired at a resolution of 15,000 and a dynamic exclusion window of 20 s was applied.

For data analysis, Xcalibur raw files were converted into the MGF format using Mascot Distliler (Matrix Science) and used directly as input files for Mascot.^[11]^ Searches were performed against an *ad hoc* protein database containing the sequence of the protein. The following parameters were set for the searches: maximum number of missed cleavages: 3; minimum peptide length 5 amino acids; variable modifications: ***r*-mS-2**-modification of cysteine (mass shift 505.049 Da), methionine oxidation (mass shift 15.99491 Da). Finally, each fragmentation spectrum was manually inspected and validated.

Denatured proteins were injected onto a C4 BEH 1.7 µm, 1.0 x 100 mm UPLC column using an Acquity I class LC (Waters, UK). Proteins were eluted with a 15 min gradient of acetonitrile (2% (*v*/*v*) to 80% (*v*/*v*)) in water with 0.1% (*v*/*v*) formic acid using a flow rate of 50 µL/min. The analytical column outlet was directly interfaced *via* an ESI source, with a time-of-flight (TOF) mass spectrometer (BioAccord, Waters, UK). Data were acquired over a *m/z* range of 300 –8000, in positive ion mode with a cone voltage of 40 V. Scans were summed together manually and deconvoluted using MaxEnt1 (Masslynx, Waters, UK).

## S7 HMT Assay Protocol for PRMT1

**Assay Principle:**

Histone methyltransferase (HMT) activity was assessed using a radiometric HotSpot™ assay format. Test compounds were pre-incubated with enzyme and substrate at room temperature, and the reaction was initiated by the addition of [³H]-*S*-adenosyl-*L*-methionine ([³H]-SAM). Incorporation of the radiolabel was quantified *via* filter binding.

**Reaction Conditions:**

Reactions were performed in a buffer containing 50 mM Tris-HCl pH 8.5, 5 mM MgCl₂, 50 mM NaCl, 1 mM TCEP, and 1% (*v*/*v*) DMSO. The standard substrate was Histone H4 (5 µM final, RBC Cat# Histone H4), and the methyl donor was [³H]-SAM (1 µM final, Revvity Cat# NET155H001MC).

**Procedure:**

Substrate was prepared in freshly made reaction buffer. PRMT1 enzyme (5 nM final, RBC Cat# HMT-11-119) was added and gently mixed. Compounds in DMSO were delivered in nanoliter volumes using an Echo 550 acoustic dispenser (LabCyte Inc.), followed by a 15 min pre-incubation at room temperature. Reactions were initiated by the addition of [³H]-SAM and incubated at 30 °C for 1 h. The reaction mixture was transferred to filter paper for detection.

**Data Analysis:**

Remaining HMT activity was expressed relative to vehicle (1% (*v*/*v*) DMSO) controls. IC₅₀ values and dose-response curve fitting were performed using GraphPad Prism (GraphPad Software) and Microsoft Excel.

**Enzyme Details:**

Recombinant human PRMT1 (GenBank Accession No. NM_001536; amino acids 2–371) was expressed in *E. coli* with an N-terminal GST tag (MW = 68.3 kDa).

## S8 Computationally-Predicted Binding Poses & Docking Procedure

The crystal structure of PRMT1 (PDB ID: 6NT2) was utilized as the receptor for docking studies. Prior to docking, the protein structure was pre-processed using AutoDockTools (ADT) to ensure suitability for simulation. This pre-processing involved several steps:

1.	Addition of Hydrogen Atoms: Polar hydrogen atoms were added to the protein structure to accurately represent hydrogen bonding interactions.

2.	Removal of Water Molecules: Crystallographic water molecules were removed to prevent potential interference with ligand binding during docking simulations.

3.	Assignment of Partial Charges: Kollman partial charges were assigned to the protein atoms to facilitate accurate electrostatic interaction calculations.

4.	Conversion to PDBQT Format: The prepared protein structure was converted to the PDBQT format, which includes information on atom types and partial charges, as required by AutoDock.

For the ligand, ***r*-mS-2,** preparation steps included:

1.	Addition of Hydrogen Atoms: All hydrogen atoms were added to the ligand to ensure proper valence representation.

2.	Assignment of Gasteiger Charges: Partial charges were assigned to the ligand atoms using the Gasteiger method.^[12]^

3.	Specification of Rotatable Bonds: The ligand’s rotatable bonds were defined to allow flexibility during docking simulations.

4.	Conversion to PDBQT Format: The ligand was converted to the PDBQT format compatible with AutoDock.

Covalent docking of ***r*-mS-2** to Cys119 was performed using AutoDock 4.2, employing a protocol optimized for accurate modelling of covalent interactions via custom ligand modification.^[13]^ The ligand was treated as flexible, allowing for conformational adaptability while maintaining coordination with the ruthenium ion. The reactive warhead of ***r*-mS-2** was manually modified in AutoDockTools to define the covalent bond formation with Cys119, ensuring proper representation of the covalent docking process. Grid box parameters were defined to encompass the Cys119 binding site, ensuring adequate sampling of ligand poses. The docking results were analysed based on binding energy scores and interaction profiles to identify the most favourable binding conformations.

# Synthesis and Characterisation

**Scheme S1**: Synthetic schemes of all reactive scaffolds studied in this work. Syntheses of: (a) **Lig-1**;(b) **r-mS-1**; (c) general scheme for **r-mS-2**, **r-mS-4** and **r-mS-5**; (d) **r-mS-3**; (e) **r-mS-6** (f) **r-mS-7** and (g) Compound **9** – non-reactive counterpart to **r-mS-2.**

## Bis(2,2’-bipyridyl)(2-chloro-*N*-1,10-phenanthrolin-5-yl-acetamide)ruthenium(II) hexafluorophosphate *r*-mS-1

Compound ***r*-mS-1** was synthesised according to a modified literature procedure.^[14–16]^ Cis dichlorobis(2,2’-bipyridyl)ruthenium(II) (89.0 mg, 184 µmol, 1.00 eq) and 2-chloro-*N*-1,10-phenanthrolin-5-yl-acetamide (**Lig-1**, 50.0 mg, 184 µmol, 1.00 eq) were suspended in dry methanol (7 mL) and refluxed for 3 h. The deep-red solution was then cooled to room temperature, filtered and the product precipitated out by addition of potassium hexafluorophosphate (339 mg, 1.84 mmol, 10 eq.). The orange-red solid was collected, washed with diethyl ether (3 x 10 mL) and allowed to dry in the dark to afford ***r*-mS-1**. Yield: 75% (95.0 mg, 139 µmol).

^1^H NMR (600 MHz, DMSO) δ = 10.83 (s, 1H, H^15^), 8.89 (dd, *J*=8.6, 1.3, 1H, H^7^), 8.85 (stack, *J*=23.1, 8.3, 4H, H^20/26^), 8.79 (dd, *J*=8.4, 1.4, 1H, H^4^), 8.63 (s, 1H, H^5^), 8.21 (stack, *J*=8.0, 1.8, 2H, H^21/27^), 8.17 (dd, *J*=5.3, 1.3, 1H, H^9^), 8.11 (stack, *J*=7.8, 4.8, 1.5, 2H, H^21/27^), 8.06 (dd, *J*=5.2, 1.4, 1H, H^2^), 7.93 (dd, *J*=8.5, 5.2, 1H, H^8^), 7.83 (stack, 3H, H^3/23/29^), 7.58 (stack, *J*=7.4, 5.8, 3.0, 4H, H^22/23/28/29^), 7.36 (stack, *J*=7.6, 5.6, 1.8, 2H, H^22/28^), 4.56 (d, *J*=2.5, 2H, H^17^) ppm.

^13^C NMR (151 MHz, DMSO) δ = 166.4 (C^16^), 156.8 (C^19/25^), 156.6 (C^19/25^), 156.5 (C^19/25^), 152.5 (C^9^), 151.5 (C^23/29^), 151.5(C^2^), 147.2 (C^12^), 144.9 (C^11^), 138.0 (C^21/27^), 137.9 (C^21/27^), 136.5 (C^4^), 133.1 (C^6^), 132.5 (C^7^), 130.1 (C^13^), 127.9 (C^22/28^), 127.8 (C^22/28^), 127.8 (C^22/28^), 126.6 (C^3^), 126.4 (C^8^), 125.9 (C^14^), 124.5 (C^20/26^), 124.4 (C^20/26^), 120.3 (C^5^), 43.4 (C^17^) ppm.

^19^F NMR (376 MHz, DMSO) δ = -70.14 (d, *J*=710.9, 12F, 2PF_6_) ppm.

UPLC-MS (ESI)^+^: *m/z* calcd for C_34_H_26_ClN_7_ORu: 324.5 [*M*-2PF_6_]^+^; found: 342.6. rt 1.08 min (100%).

HR-MS (ESI)^+^: *m/z* calcd for C_34_H_26_ClN_7_ORu: 342.5465 [*M*-2PF_6_]^+^; found: 342.5481.

**Figure S3**: ^1^H NMR spectrum (400 MHz, 298 K, DMSO-d_6_) of **r-mS-1.**

**Figure S4**: ^13^C NMR spectrum (101 MHz, 298 K, DMSO-d_6_) of **r-mS-1.**

**Figure S5**: ^19^F NMR spectrum (376 MHz, 298 K, DMSO-d_6_) of **r-mS-1**.

## Ruthenium(II)($\boldsymbol{\eta}$^6^-*p*-cymene)(2-chloro-N-1,10-phenanthrolin-5-ylacetamide)chloride, *r*-mS-2

[(*η*^6^-*p*-cymene)RuCl_2_]_2_ (90.2 mg, 147 µmol, 0.50 eq), 2-chloro-*N*-1,10-phenanthrolin-5-yl-acetamide (**Lig-1**, 80.0 mg, 294 µmol, 1.00 eq) and potassium hexafluorophosphate (54.2 mg, 294 µmol, 2.00 eq) were combined in a microwave vial and suspended in dry methanol (5 mL). The reaction mixture was sonicated for 15 min, during which a bright yellow precipitate formed. The solid was collected by filtration, washing with ice-cold water and dried *in vacuo*. The remaining filtrate was concentrated under reduced pressure and ice-cold diethyl ether was added to further precipitate the product. Once dried, both solids were combined to obtain ***r*-mS-2** as a bright yellow solid. Yield: 66% (105.1 mg, 194 µmol).

^1^H NMR (400 MHz, DMSO) δ = 10.89 (s, 1H, H^15^), 9.97 (dd, *J*=5.3, 1.1, 1H, H^9^), 9.86 (dd, *J*=5.3, 1.3, 1H, H^2^), 9.03 (dd, *J*=8.5, 1.1, 1H, H^7^), 8.92 (dd, *J*=8.4, 1.2, 1H, H^4^), 8.51 (s, 1H, H^5^), 8.23 (dd, *J*=8.5, 5.3, 1H, H^8^), 8.13 (dd, *J*=8.3, 5.2, 1H, H^3^), 6.38 – 6.32 (m, 2H, H^19^), 6.15 – 6.09 (m, 2H, H^20^), 4.57 (s, 2H, H^17^), 2.69 – 2.59 (m, 1H, H^22^), 2.17 (s, 3H, H^24^), 0.93 (d, *J*=4.2, 3H, H^23^), 0.95 – 0.89 (m, 6H, H^23^) ppm.

^13^C NMR (101 MHz, DMSO) δ = 166.4 (C^16^), 156.2 (C^9^), 155.2 (C^2^), 145.4 (C^12^), 143.2 (C^11^), 138.4 (C^4^), 134.7 (C^7^), 132.7 (C^6^), 129.5 (C^13^), 126.6 (C^3^), 126.1 (C^14^), 125.8 (C^8^), 119.9 (C^5^), 104.2 (C^21^), 102.6 (C^18^), 86.0 (C^19^), 85.9 (C^19^), 84.0 (C^20^), 83.8 (C^20^), 43.3 (C^17^), 30.4 (C^22^), 21.7 (C^23^), 18.2 (C^24^) ppm.

^19^F NMR (376 MHz, DMSO) δ = -70.10 (d, *J*=711.4, 6F, PF_6_) ppm.

UPLC-MS (ESI)^+^: *m/z* calcd for C_24_H_24_Cl_2_N_3_ORu: 542.0 [*M*-PF_6_]^+^; found: 542.1. rt 1.70 min (97%).

HR-MS (ESI)^+^: *m/z* calcd for C_24_H_24_Cl_2_N_3_ORu: 542.0340 [*M*-PF_6_]^+^; found: 542.1156.

**Figure S6**: ^1^H NMR spectrum (400 MHz, 298 K, DMSO-d_6_) of **r-mS-2**.

**Figure S7**: ^13^C NMR spectrum (101 MHz, 298 K, DMSO-d_6_) of **r-mS-2**.

**Figure S8**: ^19^F NMR spectrum (376 MHz, 298 K, DMSO-d_6_) of **r-mS-2**.

## Ferrocenecarboxaldehyde Oxime

Ferrocenecarboxaldehyde oxime was synthesised according to modified literature procedures.^[17,18]^ Ferrocenecarboxaldehyde (2.00 g, 9.34 mmol, 1.00 eq), sodium hydroxide (2.40 g, 59.8 mmol, 6.40 eq), and hydroxylamine chlorhydrate (1.40 g, 20.6 mmol, 2.20 eq) were dissolved in dry methanol (20 mL) and refluxed for 3 h. The reaction mixture was cooled to room temperature, quenched with water and stirred for a further 1 h at room temperature. The solution was extracted with CH_2_Cl_2_ (10 × 25 mL). The combined organic layers were dried over anhydrous MgSO_4_, filtered, and the solvent was evaporated under reduced pressure. The crude product was puriﬁed by column chromatography on silica with cyclohexane/ethyl acetate (2/1 →1/1) as the eluent. The product was used without further purification.

## Aminomethylferrocene, 10

Compound **10** was synthesised according to a modified literature procedure.^[17]^ Ferrocenecarboxaldehyde oxime (704 mg, 3.07 mmol) was dissolved in dry tetrahydrofuran (THF, 20 mL), and an excess of lithium aluminium hydride (LiAlH_4_, 525 mg, 13.8 mmol, 4.50 eq) was carefully added in portions. The mixture was stirred for 16 h at room temperature, following which, dry THF (10 mL) and additional equivalents of LiAlH_4_ (116 mg, 3.07 mmol, 1.00 eq) were added to the reaction mixture. The reaction solution was further stirred at room temperature for 2 h. The reaction was then quenched with diethyl ether (10 mL), water (3 x 10 mL) and aqueous sodium hydroxide (10 mL) and stirred for 15 min. Anhydrous MgSO_4_ was then added, and the slurry filtered and solvent removed under reduced pressure. The crude product was purified on a Biotage Cation Exchange Isolute SCX column and eluted with 2% ammonia/methanol. Yield: 68% (450 mg, 2.09 mmol).

^1^H NMR (400 MHz, MeOD) δ = 4.26 (s, 2H, H^2^), 4.17 (stack, 7H, H^3,4^), 3.63 (s, 2H, H^5^).

^13^C NMR (101 MHz, MeOD) δ = 87.2 (C^1^), 69.5 (C^4^), 69.3 (C^2^), 69.2 (C^3^), 41.2 (C^5^).

UPLC-MS (ESI)^+^: *m/z* calcd for C_11_H_13_FeN: 215.1 [*M*+H]^+^; found: 199.0. rt 1.19 min (100%).

**Figure S9**: ^1^H NMR spectrum (400 MHz, 298 K, MeOD-d_4_) of **10**.

**Figure S10**: ^13^C NMR spectrum (101 MHz, 298 K, MeOD-d_4_) of **10**.

## *N*-(ferrocenemethy)lchloroatemide, *r*-mS-3

Compound ***r*-mS-3** was synthesised according to a modified literature procedure.^[19]^To a stirring solution of ferrocenemethylamine (**10**, 58 mg, 270 µmol, 1.00 eq) in dry THF (4 mL) was added triethylamine (42.0 $\mu$L, 297 µmol, 1.10 eq) then chloroacetic anhydride (51.0 mg, 297 µmol, 1.10 eq) in dry THF (1 mL) at 0 °C. The reaction was allowed to slowly warm to room temperature over 1 h, after which the solvent was removed under reduced pressure. The crude product was purified on silica with a diethyl ether/cyclohexane solution (1:1) as the eluent to give ferrocenemethylchloroacetamide, ***r*-mS-3**, as a bright yellow solid. Yield: 56% (44.0 mg, 151 µmol).

^1^H NMR (600 MHz, MeOD) δ = 4.22 (s, 2H, H^3^), 4.18 (s, 5H, H^4^), 4.14 (stack, *J*=6.4, 4H, H^2^, H^8^), 4.05 (s, 2H, H^5^) ppm.

^13^C NMR (151 MHz, MeOD) δ = 168.6 (C^7^), 86.0 (C^1^), 69.6 (C^4^), 69.3 (C^2^), 69.0 (C^3^), 43.2 (C^5^), 40.0 (C^8^) ppm.

UPLC-MS (ESI)^+^: *m/z* calcd for C_13_H_14_ClFeNO: 291.6 [*M*+H]^+^; found: 291.2. rt 2.63 min (100%).

HRMS (ESI)^+^: *m/z* calcd for C_13_H_14_ClFeNO: 291.0103 [*M*+H]^+^; found: 291.0113.

**Figure S11**: ^1^H NMR spectrum (400 MHz, 298 K, MeOD-d_4_) of **r-mS-3**.

**Figure S12**: ^13^C NMR spectrum (101 MHz, 298 K, MeOD-d_4_) of **r-mS-3**.

## 2-chloro-*N*-1,10-phenanthrolin-5-yl-acetamide, Lig-1

Compound **Lig-1** was synthesised according to a modified literature procedure.^[20]^ 5-amino-1,10-phenanthroline (2.00 g, 10.2 mmol, 1.00 eq) was added to a tapped flask and cycled onto an argon-filled Schlenk line. The solid was then suspended in dry CH_2_Cl_2_ (30 mL) along with triethylamine (2.07 g, 2.86 mL, 20.5 mmol, 2.00 eq) and the suspension was cooled to 0 °C. Chloroacetyl chloride (1.27 g, 896 μL, 11.3 mmol, 1.10 eq) was added dropwise over 1.5 h, and the reaction mixture allowed to warm to room temperature overnight. The beige-brown suspension was washed with water and the organic fractions were collected and filtered. The filtrate was dried with NaSO_4_, reduced *in vacuo* and the crude was purified *via* reverse phase column chromatography (Biotage Selekt, Sfar Duo 30 g column), with acetonitrile: 0-20-50-100-50%, water/0.1% trifluoroacetic acid as the eluent. The fractions containing the desired product were combined and concentrated down and dried *in vacuo* using a VirTis SP Scientific Benchtop Pro FreezeDryer (9 L). Yield: 53 % (1.47 g, 5.41 mmol).

^1^H NMR (400 MHz, DMSO) δ = 10.55 (s, 1H, H^15^), 9.14 (dd, *J*=4.2, 1.7, 1H, H^9^), 9.06 (dd, *J*=4.3, 1.7, 1H, H^2^), 8.61 (dd, *J*=8.4, 1.7, 1H, H^7^), 8.49 (dd, *J*=8.2, 1.7, 1H, H^4^), 8.18 (s, 1H, H^5^), 7.84 (dd, *J*=8.4, 4.3, 1H, H^8^), 7.76 (dd, *J*=8.1, 4.3, 1H, H^3^), 4.52 (s, 2H, H^17^) ppm;

^13^C NMR (101 MHz, DMSO) δ = 166.1 (C^16^), 150.0 (C^9^), 149.7 (C^2^), 145.8 (C^12^), 144.0 (C^11^), 136.0 (C^4^), 131.5 (C^7^), 131.0 (C^6^), 127.9 (C^13^), 124.6 (C^14^), 123.6 (C^3^), 122.9 (C^8^), 120.8 (C^5^), 43.3 (C^17^) ppm.

UPLC-MS (ESI)^+^: *m/z* calcd for C_14_H_10_ClN_3_O: 272.7 [*M*+H]^+^; found: 272.1. rt 1.28 min (100%).

**Figure S13**: ^1^H NMR spectrum (400 MHz, 298 K, DMSO-d_6_) of **Lig-1**.

**Figure S14**: ^13^C NMR spectrum (101 MHz, 298 K, DMSO-d_6_) of **Lig-1.**

## Ruthenium(II)($\boldsymbol{\eta}$^6^-hexamethylbenzene)(2-chloro-N-1,10-phenanthrolin-5-ylacetamide)chloride, *r*-mS-4

[(*η*^6^-hexamethylbenzene)RuCl_2_]_2_ (98.4 mg, 147 µmol, 0.50 eq), 2-chloro-*N*-1,10-phenanthrolin-5-yl-acetamide (**Lig-1**, 80.0 mg, 294 µmol, 1.00 eq) and potassium hexafluorophosphate (108 mg, 589 µmol, 2.00 eq) were combined in a microwave vial and suspended in dry methanol (5 mL). The reaction mixture was sonicated for 15 min, during which a bright yellow precipitate formed. The solid was collected by filtration, washing with ice-cold water and dried *in vacuo*. The remaining filtrate was concentrated under reduced pressure and ice-cold diethyl ether was added to further precipitate the product. Once dried, both solids were combined to obtain ***r*-mS-4**. Yield: 73% (123 mg, 216 µmol).

^1^H NMR (400 MHz, DMSO) δ = 10.80 (s, 1H, H^15^), 9.40 (dd, *J*=5.3, 1.1, 1H, H^9^), 9.30 (dd, *J*=5.3, 1.2, 1H, H^2^), 8.94 (dd, *J*=8.5, 1.1, 1H, H^7^), 8.87 (dd, *J*=8.3, 1.2, 1H, H^4^), 8.50 (s, 1H, H^5^), 8.21 (dd, *J*=8.5, 5.3, 1H, H^8^), 8.12 (dd, *J*=8.2, 5.3, 1H, H^3^), 4.54 (d, *J*=0.9, 2H, H^17^), 2.12 (s, 18H, H^19^) ppm;

^13^C NMR (101 MHz, DMSO) δ = 166.4 (C^16^), 153.9 (C^9^), 153.0 (C^2^), 145.5 (C^12^), 143.3 (C^11^), 137.9 (C^4^), 134.2 (C^7^), 132.7 (C^6^), 129.3 (C^13^), 126.7 (C^3^), 126.0 (C^8^), 125.9 (C^14^), 120.0 (C^5^), 95.4 (C^18^), 43.3 (C^17^), 15.3 (C^19^) ppm;

^19^F NMR (376 MHz, DMSO) δ = -70.12 (d, *J*=711.3, 6F, PF_6_) ppm.

UPLC-MS (ESI)^+^: *m/z* calcd for C_26_H_28_Cl_2_N_3_ORu: 570.1 [*M*-PF_6_]^+^; found: 570.1. rt 1.77 min (91%).

HRMS (ESI)^+^: *m/z* calcd for C_26_H_28_Cl_2_N_3_ORu: 570.0652 [*M*-PF_6_]^+^; found: 570.0692.

**Figure S15**: ^1^H NMR spectrum (400 MHz, 298 K, DMSO-d_6_) of **r-mS-4**.

**Figure S16**: ^13^C NMR spectrum (101 MHz, 298 K, DMSO-d_6_) of **r-mS-4**.

**Figure S17**: ^19^F NMR spectrum (376 MHz, 298 K, DMSO-d_6_) of **r-mS-4**.

## Ruthenium(II)($\boldsymbol{\eta}$^6^-benzene)(2-chloro-N-1,10-phenanthrolin-5-ylacetamide)chloride, *r*-mS-5

[(*η*^6^-benzene)RuCl_2_]_2_ (73.6 mg, 147 µmol, 0.50 eq), 2-chloro-*N*-1,10-phenanthrolin-5-yl-acetamide (**Lig-1**, (80 mg, 294 µmol, 1.00 eq) and potassium hexafluorophosphate (108 mg, 589 µmol, 2.00 eq) were combined in a microwave vial and suspended in dry methanol (5 mL). The reaction mixture was sonicated for 15 min, during which a bright yellow precipitate formed. The solid was collected by filtration, washing with ice-cold water and dried *in vacuo*. The remaining filtrate was concentrated under reduced pressure and ice-cold diethyl ether was added to further precipitate the product. Once dried, both solids were combined to obtain ***r*-mS-5**. Yield: 48% (68.0 mg, 294 µmol).

^1^H NMR (400 MHz, DMSO) δ = 10.82 (s, 1H, H^15^), 10.06 (dd, *J*=5.2, 1.2, 1H, H^9^), 9.95 (dd, *J*=5.3, 1.3, 1H, H^2^), 8.97 (dd, *J*=8.4, 1.2, 1H, H^7^), 8.91 (dd, *J*=8.3, 1.2, 1H, H^4^), 8.48 (s, 1H, H^5^), 8.23 (dd, *J*=8.5, 5.2, 1H, H^8^), 8.13 (dd, *J*=8.3, 5.2, 1H, H^3^), 6.31 (s, 6H, H^18^), 4.55 (d, *J*=1.0, 2H, H^17^) ppm;

^13^C NMR (101 MHz, DMSO) δ = 166.4 (C^16^), 156.4 (C^9^), 155.5 (C^2^), 145.6 (C^12^), 143.4 (C^11^), 138.4 (C^4^), 134.8 (C^7^), 132.7 (C^13^), 129.5 (C^6^), 126.4 (C^3^), 126.3 (C^14^), 125.7 (C^8^), 119.9 (C^5^), 86.7 (C^18^), 43.3 (C^17^) ppm;

^19^F NMR (376 MHz, DMSO) δ = -70.11 (d, *J*=711.4, 6H, PF_6_) ppm.

UPLC-MS (ESI)^+^: *m/z* calcd for C_20_H_16_Cl_2_N_3_ORu: 486.0 [*M*-PF_6_]^+^; found: 487.9. rt 1.26 min (100%).

HRMS (ESI)^+^: *m/z* calcd for C_20_H_16_Cl_2_N_3_ORu: 486.9714 [*M*-PF_6_]^+^; found: 486.9713.

**Figure S18**: ^1^H NMR spectrum (400 MHz, 298 K, DMSO-d_6_) of **r-mS-5**.

**Figure S19**: ^13^C NMR spectrum (101 MHz, 298 K, DMSO-d_6_) of **r-mS-5**.

**Figure S20**: ^19^F NMR spectrum (376 MHz, 298 K, DMSO-d_6_) of **r-mS-5**.

## Osmium(II)($\boldsymbol{\eta}$^6^-*p*-cymene) dichloride, 11

Compound **11** was synthesised according to a modified literature procedure.^[21]^ A 2-5 mL microwave reactor vial fitted with a septum, was charged with sodium hexachloroosmate dihydrate (400 mg, 857 µmol, 1.00 eq) and α-phellandrene (1.00 mL, 6.21 mmol, 7.25 eq). The apparatus was then evacuated and filled with argon. Ethanol (4 mL) was added and the resulting mixture was degassed with argon for 20 min before heating to 100 °C and stirring for 3 h in a microwave reactor. After cooling to room temperature, the resulting suspension was cooled to 5 °C over 48 h. The resulting orange precipitate was collected by vacuum filtration, washed with ice-cold ethanol (2 x 10 mL) and diethyl ether (2 x 10 mL), and dried under vacuum to yield **11** as an orange solid. Yield: 37% (252 mg, 319 µmol).

^1^H NMR (400 MHz, CDCl_3_) δ = 6.17 (d, *J*=6.0, 2H), 6.02 (d, *J*=6.0, 2H), 2.92 – 2.66 (m, 1H), 2.19 (s, 3H), 1.31 – 1.23 (m, 6H);

^13^C NMR (101 MHz, CDCl_3_) δ = 92.6, 89.2, 74.1, 72.6, 31.4, 22.6, 19.6;

Attempts to obtain LC-MS data on **11** were unsuccessful due to such dimers acknowledged not to be stable under standard measurement conditions; only the monomeric Os(II)($\eta$^6^-*p*-cymene)chloride, formate and acetonitrile adducts were observed. ^1^H and ^13^C NMR spectra are consistent with reported data in literature.^[21]^

**Figure S21**: ^1^H NMR spectrum (400 MHz, 298 K, CDCl_3_) of **11**.

**Figure S22**: ^13^C NMR spectrum (101 MHz, 298 K, CDCl_3_) of **11**.

## Osmium(II)($\boldsymbol{\eta}$^6^-*p*-cymene)(2-chloro-N-1,10-phenanthrolin-5-ylacetamide)chloride, *r*-mS-6

[(*η*^6^-*p*-cymene)OsCl_2_]_2_ (**11**, 102 mg, 129 µmol, 0.50 eq), 2-chloro-*N*-1,10-phenanthrolin-5-yl-acetamide (**Lig-1**, 70 mg, 258 µmol, 1.00 eq) and potassium hexafluorophosphate (94.8 mg, 294 µmol, 2.00 eq) were combined in a microwave vial and suspended in dry methanol (5 mL). The reaction mixture was sonicated for 15 min, during which a bright yellow precipitate formed. The solid was collected by filtration, washing with ice-cold water and dried *in vacuo*. The remaining filtrate was concentrated under reduced pressure and ice-cold diethyl ether was added to further precipitate the product. Once dried, both solids were combined to obtain ***r*-mS-6**. Yield: 66% (108 mg, 171 µmol).

^1^H NMR (400 MHz, DMSO) δ = 10.86 (s, 1H, H^15^), 9.91 (d, *J*=5.3, 1H, H^9^), 9.80 (d, *J*=5.2, 1H, H^2^), 9.01 (d, *J*=8.5, 1H, H^7^), 8.92 (d, *J*=8.3, 1H, H^4^), 8.59 (s, 1H, H^5^), 8.31 – 8.15 (m, 1H, H^8^), 8.15 – 8.00 (m, 1H, H^3^), 6.68 – 6.46 (m, 2H, H^19^), 6.43 – 6.18 (m, 2H, H^20^), 4.57 (s, 2H, H^17^), 2.23 (s, 3H, H^24^), 0.86 (s, 6H, H^23^) ppm.

^13^C NMR (101 MHz, DMSO) δ = 166.4 (C^16^), 156.1 (C^9^), 155.1 (C^2^), 146.6 (C^12^), 144.2 (C^11^), 138.6 (C^4^), 134.9 (C^7^), 133.1 (C^6^), 129.7 (C^13^), 127.2 (C^3^), 126.5 (C^8^), 126.3 (C^14^), 119.9 (C^5^), 95.4 (C^21^), 95.0 (C^18^), 77.6 (C^19^), 77.5 (C^19^), 74.5 (C^20^), 74.3 (C^20^), 43.3 (C^17^), 30.5 (C^22^), 21.9 (C^23^), 18.1 (C^24^) ppm.

^19^F NMR (376 MHz, DMSO) δ = -70.12 (d, *J*=711.3, 6H, PF_6_) ppm.

NB: The signal for the isopropyl CH (H^22^) is stacked with the DMSO peak in the ^1^H NMR at 2.50 ppm. However the signal for the carbon (C^22^) is visible as expected in the ^13^C NMR at 30.5 ppm.

UPLC-MS (ESI)^+^: *m/z* calcd for C_14_H_10_ClN_3_OOs: 632.1 [*M*-PF_6_]^+^; found: 632.0. rt 1.76 min (100%).

HRMS (ESI)^+^: *m/z* calcd for C_14_H_10_ClN_3_OOs 631.6060 [M-PF_6_]^+^; found: 631.6056.

**Figure S23**: ^1^H NMR spectrum (400 MHz, 298 K, DMSO-d_6_) of **r-mS-6**.

**Figure S24**: ^13^C NMR spectrum (101 MHz, 298 K, DMSO-d_6_) of **r-mS-6**.

**Figure S25**: ^19^F NMR spectrum (376 MHz, 298 K, DMSO-d_6_) of **r-mS-6**.

## Iridium(III)($\boldsymbol{\eta}$^5^-cyclopentadienyl)(2-chloro-N-1,10-phenanthrolin-5-ylacetamide)chloride, *r*-mS-7

[(*η*^5^-cyclopentadienyl)IrCl_2_]_2_ (50.0 mg, 93.7 µmol, 0.5 eq.), 2-chloro-*N*-1,10-phenanthrolin-5-yl-acetamide (**Lig-1**, 50.9 mg, 187 µmol, 1.00 eq.) and suspended in dry methanol (5 mL). A methanolic solution of KPF_6_ (69.0 mg, 375 µmol, 2.00 eq.) was then added, and the reaction mixture stirred for 16 h, after which a bright yellow-orange precipitate formed. The solid was collected by filtration, washing with ice-cold water and dried *in vacuo*. The remaining filtrate was concentrated under reduced pressure and ice-cold diethyl ether was added to further precipitate the product. Once dried, both solids were combined to obtain ***r*-mS-7**. Yield: 36% (52.0 mg, 66.7 µmol).

^1^H NMR (400 MHz, DMSO) δ = 10.90 (s, 1H, H^15^), 9.42 (d, *J*=5.2, 1H, H^9^), 9.33 (d, *J*=5.2, 1H, H^2^), 9.02 (d, *J*=8.5, 1H, H^7^), 8.96 (d, *J*=8.2, 1H, H^4^), 8.58 (s, 1H, H^5^), 8.27 (dd, *J*=8.5, 5.2, 1H, H^8^), 8.17 (dd, *J*=8.3, 5.2, 1H, H^3^), 4.57 (s, 2H, H^17^), 1.73 (s, 15H, H^19^) ppm;

^13^C NMR (101 MHz, DMSO) δ = 166.5 (C^16^), 152.4 (C^9^), 151.4 (C^2^), 146.6 (C^12^), 144.3 (C^11^), 138.8 (C^7^), 135.1 (C^4^), 133.2 (C^6^), 129.8 (C^13^), 127.6 (C^3^), 127.0 (C^8^), 126.6 (C^14^), 120.4 (C^5^), 89.2 (C^18^), 43.3 (C^17^), 8.2 (C^19^) ppm;

^19^F NMR (376 MHz, DMSO) δ = -70.12 (d, *J*=711.3, 6H, PF_6_) ppm.

HR-MS (ESI)^+^: *m/z* calcd for C_14_H_10_ClIrN_3_O: 634.6010 [M-PF_6_]^+^; found: 634.6012.

**Figure S26**: ^1^H NMR spectrum (400 MHz, 298 K, DMSO-d_6_) of **r-mS-7**.

**Figure S27**: ^13^C NMR spectrum (101 MHz, 298 K, DMSO-d_6_) of **r-mS-7**.

**Figure S28**: ^19^F NMR spectrum (376 MHz, 298 K, DMSO-d_6_) of **r-mS-7**.

## Ruthenium(II)($\boldsymbol{\eta}$^6^-*p*-cymene)(5-methyl-1,10-phenanthroline)chloride, compound 9

[(*η*^6^-*p*-cymene)RuCl_2_]_2_ (89.9 mg, 147 µmol, 0.50 eq), 5-methyl-1,10-phenanthroline (57.0 mg, 293 µmol, 1.00 eq) and potassium hexafluorophosphate (108 mg, 587 µmol, 2.00 eq) were combined in a microwave vial and suspended in dry methanol (5 mL). The reaction mixture was sonicated for 15 min, during which a bright yellow precipitate formed. The solid was collected by filtration, washing with ice-cold water and dried *in vacuo*. The remaining filtrate was concentrated under reduced pressure and ice-cold diethyl ether was added to further precipitate the product. Once dried, both solids were combined to obtain compound **9** as a bright yellow solid. Yield: 34% (46 mg, 99 µmol).

^1^H NMR (600 MHz, DMSO) δ = 9.93 (dd, *J*=5.2, 1.4, 1H, H^9^), 9.85 (dd, *J*=5.2, 1.4, 1H, H^2^), 8.94 (dd, *J*=8.5, 1.3, 1H, H^7^), 8.80 (dd, *J*=8.3, 1.4, 1H, H^4^), 8.18 (dd, *J*=8.4, 5.2, 1H, H^8^), 8.11 (dd, *J*=8.1, 5.2, 1H, H^3^), 8.10 (s, 1H, H^5^), 6.39 – 6.27 (m, 2H, H^17^), 6.16 – 6.01 (m, 2H, H^18^), 2.83 (s, 3H, H^15^), 2.60 (hept, *J*=7.0, 1H, H^20^), 2.17 (s, 3H, H^22^), 0.93 – 0.85 (m, 6H, H^21^) ppm;

^13^C NMR (151 MHz, DMSO) δ = 155.6 (C^9^), 155.1 (C^2^), 145.4 (C^12^), 144.4 (C^11^), 137.9 (C^7^), 136.1 (C^4^), 135.6 (C^6^), 130.2 (C^14^), 129.7 (C^13^), 126.4 (C^8^), 126.1 (C^5^), 126.0 (C^3^), 103.9 (C^16^), 102.8 (C^19^), 86.1 (C^17^), 86.0 (C^17^), 83.8 (C^18^), 83.7 (C^18^), 30.4 (C^20^), 21.7 (C^21^), 21.6 (C^21^), 18.2 (C^22^), 18.2 (C^15^) ppm;

^19^F NMR (376 MHz, DMSO) δ = -70.13 (d, *J*=711.4, 6H, PF_6_) ppm.

UPLC-MS (ESI)^+^: *m/z* calcd for C_23_H_24_ClN_2_ORu: 465.0 [*M*-PF_6_]^+^; found: 465.0. rt 1.83 min (100%).

**Figure S29**: ^1^H NMR spectrum (400 MHz, 298 K, DMSO-d_6_) of compound **9**.

**Figure S30**: ^13^C NMR spectrum (101 MHz, 298 K, DMSO-d_6_) of compound **9**.

**Figure S31**: ^19^F NMR spectrum (376 MHz, 298 K, DMSO-d_6_) of compound **9**.

# S9 *r*-mS Scaffold Nomenclature and Structures

| Name | Structure |
| --- | --- |
| *r*-mS-1 |  |
| *r*-mS-2 |  |
| *r*-mS-3 |  |
| Lig-1 |  |
| *r*-mS-4 |  |
| *r*-mS-5 |  |
| *r*-mS-6 |  |
| *r*-mS-7 |  |
| 9 |  |
| 10 |  |
| 11 |  |

**Table S2**: **r-mS Scaffolds**. Table of all compounds, intermediates and control synthesised for this study.

# S10 PMI Plot of *r*-mS

**Figure S32**: PMI analysis of metal complex scaffolds (octahedral Ru scaffold = blue; pseudo-octahedral Ru piano stool complex = pink; ferrocene = green), compared with PMI scores of our internal organic fragment library (grey).

# S11 Stability of *r*-mS

**Figure S33**: **Stability of r-mS-1**. Stacked UPLC chromatograms (260 nm) of **r-mS-1** (10 $\mu$M) in RIPA lysis buffer pH 8.0 with acetaminophen (25 $\mu$M) as an internal standard from t=0 to t=60 min. Bar chart shows ratio [%] of **r-mS-1** to internal standard at various timepoints for a total of t=60 min in RIPA lysis buffer, calculated using the first equation on page 11.

**Figure S34**: **Stability of r-mS 2**. Stacked UPLC chromatograms (260 nm) of **r-mS-2** (10 $\mu$M) in RIPA lysis buffer pH 8.0 with caffeine (25 $\mu$M) as an internal standard from t=0 to t=60 min. Peak at retention time = 2.07 min corresponds to intact chlorido **r-mS-2** (m/z 542.0). Peak at retention time = 1.70 min corresponds to **r-mS-2-**derived chloride-loss species (m/z 507.0, [M-PF_6_]^+^ without monodentate chloride ligand). Minor peak at retention time = 2.02 min corresponds to formate-bound **r-mS-2**. Peak at retention time = 1.85 min corresponds to caffeine internal standard. Bar chart shows ratio [%] of **r-mS-2** to internal standard at various timepoints for a total of t=60 min in RIPA lysis buffer, calculated using the first equation on page 11.

**Figure S35**: **Stability of r-mS 3**. Stacked UPLC chromatograms (260 nm) of **r-mS-3** (10 $\mu$M) in RIPA lysis buffer pH 8.0 with caffeine (25 $\mu$M) as an internal standard from t=0 to t=60 min. Bar chart shows ratio [%] of **r-mS-3** to internal standard at various timepoints for a total of t=60 min in RIPA lysis buffer, calculated using the first equation on page 11.

***r*-mS-1**

**Figure S36**: **Stability of r-mS**. ^1^H NMR spectrum stackplot of **r-mS-1** over 1 month (MeOD-d_4_, 600 MHz, 298 K).

***r*-mS-2**

**Figure S37**: **Stability of r-mS**. ^1^H NMR spectrum stackplot of **r-mS-2** over 2 months in (DMSO-d_6_, 400 MHz, 298 K).

***r*-mS-3**

**Figure S38**: **Stability of r-mS**. ^1^H NMR spectrum stackplot of **r-mS-3** over 2 months (DMSO-d_6_, 400 MHz, 298 K).

# S12 3-Point Concentration Data for First Generation *r*-mS

**Figure S39**: Stacked volcano plots showing interactions between **r-mS-1**, **r-mS-2**, **r-mS-3** and **Lig-1** with a cysteine-containing peptide at three treatment conditions (12.5, 50 and 200 µM). Liganding events are seen to increase in number with increasing treatment concentration, validating the readout of the chemoproteomic platform.

# S13 Reactivity between *r*-mS-2 and Amino Acids

**Figure S40**: **Incubation of r-mS-2 with amino acids**. UV traces (260 nm) upon incubation of **r-mS-2** with various amino acids in RIPA lysis buffer at pH 8.0 (2% ((v/v)) DMSO) at 25 °C for 2 h in the dark.

**Figure S41**: Incubation of **r-mS-2** with amino acids blank - UPLC chromatogram at 260 nm of **r-mS-2** and caffeine as an internal standard in RIPA lysis buffer at pH 8.0 (2% ((v/v)) DMSO) at 25 °C.

**Figure S42**: **Incubation of r-mS-2 with amino acids – cysteine.** **A**. UPLC chromatogram stackplot at 290 nm of **r-mS-2**-cysteine incubated at 25 °C from t=0 to t=90, showing formation of **r-mS-2**-cysteine-associated species (purple), and **B**. zoomed version of chromatogram stack A.

**Figure S43**: **Incubation of r-mS, Lig-1 and non-reactive control compound 9, with cysteine.** Compounds were incubated in a 1:1 molar ratio with L-cysteine (500 µM each) in RIPA buffer (pH 8.0, 2% (v/v) DMSO) and monitored by UPLC–MS at 10 min intervals over 90 min. Plots show reciprocal concentration (1/[Compound]ₜ) versus time, with linear regression fits (95% confidence interval, grey). Derived half-lives are tabulated for each compound.

# S14 Structural Feature Enrichment Analysis

**Figure S44:** Feature enrichment analysis comparing reactive metallo-scaffolds to organic ligand hits at 50 μM concentration. **a**. Heatmap showing the statistical significance (-log_10_(p-value)) of structural feature differences between reactive metallo-scaffolds (**r-mS-1**, **r-mS-2**, **r-mS-3**) and the organic ligand (**Lig-1**) for liganded cysteines. Statistical significance was calculated in a non-parametric manner, by applying Mann-Whitney U-tests. The "All metals" column represents the combined comparison of all three reactive metallo-scaffolds versus **Lig-1**, while individual columns show pairwise comparisons between each reactive metallo-scaffolds and **Lig-1**. **b**. Violin plots showing the distribution of nine structural features that best distinguish reactive metallo-scaffolds from **Lig-1**. These features were selected from the collective analysis shown in panel a based on their statistical significance and biological relevance. p-values from Mann-Whitney U tests comparing all reactive metallo-scaffolds versus **Lig-1** are displayed. These selected features highlight the most important structural determinants driving the differential cysteine targeting preferences between reactive metallo-scaffolds and the organic ligand.

S15 8-Point Concentration-Response Curves: ***r*-mS** and PRMT1_Cys119

**Figure S45**: 8-point concentration response curves from chemoproteomic profiling show Cys119 engagement by **r-mS**, with **r-mS-4** included.

# S16 *p*TE50 Values of Second Generation *r*-mS

**Table S3:** *p*TE_50_ values of ***r*-mS-2** and its second-generation *r-*mS. *p*TE_50_ curves were fit using GraphPad Prism using the “log(inhibitor) vs. response - Variable slope (four parameters)” method with no applied constraints.

| Compound | Structure | *p*TE_50_ | Top | Bottom |
| --- | --- | --- | --- | --- |
| *r*-mS-2 |  | 5.2 ± 0.02 | 94.16 | 3.670 |
| *r*-mS-4 |  | N.D. | - | - |
| *r*-mS-5 |  | 5.1 ± 0.13 | 121.8 | 54.19 |
| *r*-mS-6 |  | 4.8 ± 0.09 | 107.5 | 33.38 |
| *r*-mS-7 |  | 4.5 ± 0.07 | 109.0 | 7.747 |

# S17 Intact Protein LC-MS Data of PRMT1

**Figure S46:** Intact mass spectrum of purified PRMT1.

**Figure S47:** Intact mass spectrum of PRMT1 covalently-modified by **r-mS-2**.

**Figure S48:** Fragmentation spectra of m/z 695.9 showing location of modified cysteine. Inset: zoomed MS region showing the characteristic ruthenium isotopic pattern of the **r-mS-2**-modified peptide. The Ru isotopic envelope was resolved in the peptide-level LC-MS/MS data, but not in the intact protein MS spectra shown in Figures S46–S47.

# S18 Electrostatic Representation of *r*-mS-2-docked PRMT1

**Figure S49**: Electrostatic representation of PRMT1 (top) with docked **r-mS-2** in SAM-binding domain superimposed (bottom). Negative charge – red; positive charge – blue. PDB: 6NT2.

# References

[1]	A. S. Romanov, D. V. Muratov, A. R. Kudinov, “High-yield synthesis of osmium complexes [(η6-C6H6)OsCl2]2, [CpOs(η6-C6H6)]+ and CpOs(η5-cyclohexadienyl)” *J. Organomet. Chem.* **2013**, *724*, 177–179.

[2]	G. S. Biggs, E. E. Cawood, A. Vuorinen, W. J. McCarthy, H. Wilders, I. G. Riziotis, A. J. van der Zouwen, J. Pettinger, L. Nightingale, P. Chen, A. J. Powell, D. House, S. J. Boulton, J. M. Skehel, K. Rittinger, J. T. Bush, “Robust proteome profiling of cysteine-reactive fragments using label-free chemoproteomics” *Nat. Commun.* **2025**, *16*, 1–14.

[3]	M. Kuljanin, D. C. Mitchell, D. K. Schweppe, A. S. Gikandi, D. P. Nusinow, N. J. Bulloch, E. V. Vinogradova, D. L. Wilson, E. T. Kool, J. D. Mancias, B. F. Cravatt, S. P. Gygi, “Reimagining high-throughput profiling of reactive cysteines for cell-based screening of large electrophile libraries” *Nat. Biotechnol.* **2021**, *39*, 630–641.

[4]	M. Wirth, W. H. B. Sauer, “Bioactive Molecules: Perfectly Shaped for Their Target?” *Mol. Inform.* **2011**, *30*, 677–688.

[5]	N. M. O’Boyle, M. Banck, C. A. James, C. Morley, T. Vandermeersch, G. R. Hutchison, “Open Babel: An open chemical toolbox” *J. Cheminformatics* **2011**, *3*, 33.

[6],	“PROPKA3: Consistent Treatment of Internal and Surface Residues in Empirical pKa Predictions | Journal of Chemical Theory and Computation,” can be found under https://pubs.acs.org/doi/10.1021/ct100578z(accessed 23 October 2025), **n.d.**

[7]	V. Le Guilloux, P. Schmidtke, P. Tuffery, “Fpocket: An open source platform for ligand pocket detection” *BMC Bioinformatics* **2009**, *10*, 168.

[8]	M. E. H. White, J. Gil, E. W. Tate, “Proteome-wide structural analysis identifies warhead- and coverage-specific biases in cysteine-focused chemoproteomics” *Cell Chem. Biol.* **2023**, *30*, 828-838.e4.

[9]	P. J. A. Cock, T. Antao, J. T. Chang, B. A. Chapman, C. J. Cox, A. Dalke, I. Friedberg, T. Hamelryck, F. Kauff, B. Wilczynski, M. J. L. de Hoon, “Biopython: freely available Python tools for computational molecular biology and bioinformatics” *Bioinforma. Oxf. Engl.* **2009**, *25*, 1422–1423.

[10],	“PRMT1 protein arginine methyltransferase 1 [Homo sapiens (human)] - Gene - NCBI,” can be found under https://www.ncbi.nlm.nih.gov/gene/3276(accessed 25 October 2025), **n.d.**

[11],	“Probability‐based protein identification by searching sequence databases using mass spectrometry data - Perkins - 1999 - ELECTROPHORESIS - Wiley Online Library,” can be found under https://analyticalsciencejournals.onlinelibrary.wiley.com/doi/abs/10.1002/(SICI)1522-2683(19991201)20:18%3C3551::AID-ELPS3551%3E3.0.CO;2-2?casa_token=dVwxRLZgfqUAAAAA%3AmdfzTGC9EgFhrsLxxEJECqIz6qV1bpsOxsPK-X99f3Dul31EYutNQVs5NvuaVoograI4IKORIJkdmMo(accessed 25 October 2025), **n.d.**

[12]	J. Gasteiger, M. Marsili, “A new model for calculating atomic charges in molecules” *Tetrahedron Lett.* **1978**, *19*, 3181–3184.

[13]	G. M. Morris, H. Ruth, W. Lindstrom, M. F. Sanner, R. K. Belew, D. S. Goodsell, A. J. Olson, “AutoDock4 and AutoDockTools4: Automated docking with selective receptor flexibility” *J. Comput. Chem.* **2009**, *30*, 2785–2791.

[14]	F. N. Castellano, J. D. Dattelbaum, J. R. Lakowicz, “Long-Lifetime Ru(II) Complexes as Labeling Reagents for Sulfhydryl Groups” *Anal. Biochem.* **1998**, *255*, 165–170.

[15]	A. Fedorova, A. Chaudhari, M. Y. Ogawa, “Photoinduced electron-transfer along α-helical and coiled-coil metallopeptides” *J. Am. Chem. Soc.* **2003**, *125*, 357–362.

[16]	A. Fedorova, M. Y. Ogawa, “Site-specific modification of de novo designed coiled-coil polypeptides with inorganic redox complexes” *Bioconjug. Chem.* **2002**, *13*, 150–154.

[17]	J. Hess, M. Patra, V. Pierroz, B. Spingler, A. Jabbar, S. Ferrari, R. B. Gasser, G. Gasser, “Synthesis, Characterization, and Biological Activity of Ferrocenyl Analogues of the Anthelmintic Drug Monepantel” *Organometallics* **2016**, *35*, 3369–3377.

[18]	J. Lasri, A. S. Elsherbiny, N. E. Eltayeb, M. Haukka, M. E. El-Hefnawy, “Synthesis and characterization of ferrocene-based Schiff base and ferrocenecarboxaldehyde oxime and their adsorptive removal of methyl blue from aqueous solution” *J. Organomet. Chem.* **2018**, *866*, 21–26.

[19]	M. C. Martos-Maldonado, I. Quesada-Soriano, F. García-Maroto, A. Vargas-Berenguel, L. García-Fuentes, “Ferrocene labelings as inhibitors and dual electrochemical sensors of human glutathione S-transferase P1-1” *Bioorg. Med. Chem. Lett.* **2012**, *22*, 7256–7260.

[20]	E. Belhadj, A. El-Ghayoury, T. Cauchy, M. Allain, M. Mazari, M. Sallé, “Tetrathiafulvalene-Based Phenanthroline Ligands: Synthesis, Crystal Structures, and Electronic Properties” *Eur. J. Inorg. Chem.* **2014**, *2014*, 3912–3919.

[21]	H. Werner, K. Zenkert, “Aromaten(phosphan)metall-Komplexe: XIII. osmium(II)- und osmium(O)-komplexe mit p-cymen als aromatischem liganden” *J. Organomet. Chem.* **1988**, *345*, 151–166.
